# *Salmonella* Typhimurium infection disrupts but continuous feeding of *Bacillus* based probiotic restores gut microbiota in infected hens

**DOI:** 10.1186/s40104-020-0433-7

**Published:** 2020-03-23

**Authors:** Samiullah Khan, Kapil K. Chousalkar

**Affiliations:** School of Animal and Veterinary Sciences, The University of Adelaide, Roseworthy, South Australia 5371 Australia

**Keywords:** 16S rRNA sequencing, Chicken gut microbiota, Gut metabolites, *Salmonella* Typhimurium, Strategic feeding of probiotic

## Abstract

**Background:**

The gut microbiota plays an important role in the colonisation resistance and invasion of pathogens. *Salmonella* Typhimurium has the potential to establish a niche by displacing the microbiota in the chicken gut causing continuous faecal shedding that can result in contaminated eggs or egg products. In the current study, we investigated the dynamics of gut microbiota in laying chickens during *Salmonella* Typhimurium infection. The optimisation of the use of an infeed probiotic supplement for restoration of gut microbial balance and reduction of *Salmonella* Typhimurium load was also investigated.

**Results:**

*Salmonella* infection caused dysbiosis by decreasing (FDR < 0.05) the abundance of microbial genera, such as *Blautia*, *Enorma*, *Faecalibacterium*, *Shuttleworthia*, *Sellimonas*, *Intestinimonas* and *Subdoligranulum* and increasing the abundance of genera such as *Butyricicoccus*, *Erysipelatoclostridium*, *Oscillibacter* and *Flavonifractor*. The higher *Salmonella* Typhimurium load resulted in lower (*P* < 0.05) abundance of genera such as *Lactobacillus*, *Alistipes*, *Bifidobacterium*, *Butyricimonas*, *Faecalibacterium* and *Romboutsia* suggesting *Salmonella* driven gut microbiota dysbiosis. Higher *Salmonella* load led to increased abundance of genera such as *Caproiciproducens*, *Acetanaerobacterium*, *Akkermansia*, *Erysipelatoclostridium*, *Eisenbergiella*, *EscherichiaShigella* and *Flavonifractor* suggesting a positive interaction of these genera with *Salmonella* in the displaced gut microbiota. Probiotic supplementation improved the gut microbiota by balancing the abundance of most of the genera displaced by the *Salmonella* challenge with clearer effects observed with continuous supplementation of the probiotic. The levels of acetate and butyrate in the faeces were not affected (*P* > 0.05) by *Salmonella* challenge and the butyrate level was increased by the continuous feeding of the probiotic. Probiotic supplementation in *Salmonella* challenged chickens resulted in higher level of propionate. Continuous probiotic supplementation decreased (*P* < 0.05) the overall mean load of *Salmonella* in faeces and had a significant effect on *Salmonella* load reduction in internal organs.

**Conclusions:**

*Salmonella* challenge negatively impacts the diversity and abundance of many gut microbial genera involved in important functions such as organic acid and vitamin production. Strategic feeding of a *Bacillus* based probiotic helps in restoring many of the microbial genera displaced by *Salmonella* Typhimurium challenge.

## Background

The chicken gut microbiome is composed of multiple microorganisms and their genetic materials. These microorganisms (microbiota) are involved in functions that are critical to bird health and performance. The gut microbiota help in digestion and metabolism [1], regulation of enterocytes [2], vitamin synthesis and development and regulation of the host immune system [3]. The chicken gut microbiota is mainly composed of the phyla Proteobacteria, Bacteroidetes, Actinobacteria and Firmicutes [4]. The host gut microbiota is affected by multiple factors such as disease, diet, husbandry conditions and age [5]. *Salmonella* Typhimurium causes clinical disease in many animals and humans; however, chickens are often asymptomatic carriers. Pathogenic *Salmonella* present in laying production systems often result in gastroenteritis in humans after the consumption of contaminated food [6]. In the chicken gut, *Salmonella* elicits inflammation through the activation of *Salmonella* pathogenicity island 1 (SPI1) for encoding the type III secretion system [7]. In the inflamed gut, motility allows *Salmonella* Typhimurium to utilize available nutrients for its enhanced growth [8]*.* To escape nutrient limitation caused by the intestinal microbiota, *Salmonella* uses specific metabolic traits for the utilisation of compounds that are not metabolized by gut microbiota [9].

Gram-negative bacteria dominate the gut at an early age, while gram positive Firmicutes, particularly Clostridia taxa, become more prominent at later ages [10]. A previous study demonstrated a negative correlation between Enterobacteriaceae and Lachnospiraceae, Ruminococcaceae, Erysipelotrichaceae and Peptostreptococcaceae in *Salmonella* Enteritidis challenged chicks [11], thus causing gut dysbiosis. Gut dysbiosis results from microbial imbalance due to impaired microbiota [12]. The mechanism by which the gut microbiome affects pathogen colonisation is partly mediated by the production of short-chain fatty acids (SCFAs) that are the metabolites of bacterial fermentation of undigested dietary fibre [13]. SCFAs activate G protein-coupled receptors (GPCRs) including free fatty-acid receptors 2 and 3 (FFAR2 and FFAR3) [14], inhibit histone deacetylases [15] and provide energy to enterocytes [16]. Although the roles of GPCRs (e.g. FFAR3/GPR41 and FFAR2/GPR43) are not well established, they have been implicated in the regulation of leukocytes [17] and leptin production [18] in murine models. GPR41 and GPR43 play a role in lowering body weight through the down-regulation of leptin mRNA [18]. Previous research in broiler chickens showed that the activation of GPR41 and GPR43 by gut microbiota derived SCFAs resulted in the production of Glucagon-like peptide-1 (GLP-1), which supressed lipid accumulation in the liver [19]. Therefore, the host microbiome constitutes an attractive target for manipulation, as it can be modified for pathogen colonisation resistance to reduce disease risk.

To strengthen and improve the gut microbiota composition in chickens, pre- and pro- biotics are often supplemented as a part of the feeding regimen. Prebiotics are host non-digestible complex carbohydrates that help to increase the resident gut microbiota through fermentation. Examples of prebiotics are pectin, xylooligosaccharides, galactooligosaccharides, fructooligosaccharides and inulin. Probiotics are live microbial feed supplements that beneficially affect the host by improving its intestinal microbial balance [20]. The representative bacterial genera in probiotics include *Lactobacillus*, *Bifidobacterium*, *Enterococcus*, *Streptococcus* and *Bacillus*. Apart from gram-positive bacteria, some probiotics are also composed of yeast and moulds. Some of the proposed functions of probiotics include competitive exclusion [21], antagonism [22], bacterial interference [23], barrier effect [24], modulation of host immune system [25] and colonization resistance [26]. These actions are achieved mainly through bacteria-bacteria and host-bacteria interactions. The bacteria-bacteria interactions result in the production of SCFAs [27], modification of redox potential [28], production of antimicrobial compounds, competition for epithelial receptors, quorum sensing [29] and production of an ecosystem harmful for pathogenic organisms. The reduced luminal pH due to organic acids restricts the growth of many pathogens. Probiotic bacteria secrete enzymes that hydrolyse bacterial toxins and modify toxin receptors [30]. Attachment of probiotic bacteria to cell surface receptors of enterocytes initiates signalling events that result in the synthesis of cytokines [31] and stimulation of toll-like receptors [32].

In laying hens, probiotics are generally used as feed supplements for improving flock performance and egg quality [33, 34]. From the food safety perspective, *Salmonella* is an important foodborne pathogen that is often present in the gut of chickens. *Salmonella* reduction in layers, for the production of safer egg and egg products, has always been a priority for the egg industry. In Australia, *Salmonella* Typhimurium has been responsible for the majority of the egg related foodborne outbreaks [6]. The supplemental use of probiotics lowers the incidence of *Salmonella* in poultry production [35]. Given the longer commercial life span of egg laying hens, in order to achieve the cost-effective reduction in *Salmonella* shedding, it is critical to optimise the use of probiotics and to understand the dynamics of gut microbiota during probiotic treatment. Previous studies of probiotics use for *Salmonella* control in laying chickens have mainly focused either on young chicks, using different serovars of *Salmonella* or have used a short duration trial where the effect of *Salmonella* was not tested on gut microbiota dysbiosis at different time-points while chickens were laying [36–38]. In this study, we raised *Salmonella* free birds to understand the role of *Salmonella* Typhimurium in gut microbiota dysbiosis and its subsequent restoration through the use of a *Bacillus* based probiotic in laying chickens from point of laying until 30 weeks of age. Based on the role of microbiota in the clearance of gut pathogens, we hypothesised that, if used strategically, a *Bacillus* based probiotic could be effective in positively modulating the microbiota for gut health during *Salmonella* Typhimurium infection.

## Methods

### Ethics approval

All experimental work was approved by the Animal Ethics Committee at The University of Adelaide under approval number S-2017-080 in accordance with the guidelines specified in “Australian code for the care and use of animals for scientific purposes, 8th edition (2013)”.

### Rearing of laying chickens

Eggs from an Isa-Brown parent breeder flock were obtained from a hatchery, fumigated and hatched at the School of Animal and Veterinary Sciences. Meconium samples were tested through standard culture methods for the presence of *Salmonella* spp. (if any). Before placement of day-old laying chicks, the rearing facility was tested for the presence of *Salmonella* spp. The day-old female chicks were divided into six treatment groups (7 chickens in each treatment group), reared in pens until week 14 and then transferred into individual cages. The treatment groups were: negative control (NC), *Salmonella* challenge (SX), continuous probiotic supplemented and *Salmonella* challenge (CPX), continuous probiotic supplemented control (CPC), intermittent probiotic supplemented and *Salmonella* challenge (IPX) and intermittent probiotic control (IPC). The feeding regime was as per the protocol of the ISA General Management Guide. Before adding the probiotic, the feed was fumigated and regularly tested for the presence of *Salmonella*. For the probiotic-supplemented groups, 1 g of *Bacillus* based probiotic (*Bacillus subtilis* DSM 32324, *Bacillus subtilis* DSM 32325 and *Bacillus amyloliquefaciens*) was mixed with 1 kg of fumigated feed. The intermittent probiotic supplemented groups were on the probiotic supplement for alternate 4 weeks (4 weeks ON/OFF strategy). Faeces from all the treatment groups were tested fortnightly for *Salmonella* isolation until the specific group chickens were challenged with *Salmonella* Typhimurium. At 18 weeks of age, pullets from the selected groups were orally inoculated with 10^6^ colony forming units (CFUs) per mL of *Salmonella* Typhimurium phage type 9, while the control groups received phosphate buffered saline (PBS). For the preparation of bacterial inoculum, *Salmonella* Typhimurium was grown on xylose lysine deoxycholate (XLD; ThermoFisher Scientific, Australia) agar and a single colony was subcultured in Luria-Bertani (LB) broth. The inoculum was prepared by re-suspending the washed bacterial pellet in PBS. Ten-fold serial dilutions of the original inoculum were plated onto XLD to confirm the CFU received by the individual chickens.

### Faecal shedding profile of *Salmonella* Typhimurium challenged chickens

Individual chickens were monitored for the faecal shedding profile of *Salmonella* Typhimurium by sampling the faeces on days 3, 5 and 7 and then weeks 2, 4, 6, 8, 10 and 12 post-challenge. Fresh faecal samples were collected in sterile zip lock bags from individual chickens including the control groups. Faecal samples were also collected in 1.5 mL and 5 mL tubes and stored at − 80 °C until used for microbial DNA extraction and quantification of SCFAs, respectively. The SCFAs analysis was performed on samples collected at weeks 1, 4, 8 and 12 post-challenge. A miniaturized most probable number (mMPN) method was used for the enumeration of *Salmonella* Typhimurium in individual positive faecal samples. The mMPN method was originally developed by the USDA-FDA, validated on chicken faecal samples for *Salmonella* enumeration [39] and has been used frequently in similar studies [40, 41]. The bacterial culture and mMPN procedures were performed following the methods previously described [40].

### Processing of eggs for *Salmonella* enumeration

Once the chickens were in lay, eggs from all the treatment groups were aseptically collected every fortnight in Whirl Pack plastic bags and processed for the enumeration of *Salmonella* Typhimurium on the eggshell surface and in egg internal contents following the methods previously described [41, 42]. An mMPN was performed on the samples positive for *Salmonella* Typhimurium.

### Short chain fatty acids quantification in faeces

The faecal samples stored at − 80 °C (≤ 3 month-old samples) were processed for SCFAs (acetate, propionate and butyrate) quantification using gas chromatography (Hewlett-Packard6890; Palo Alto, CA, USA) equipped with a BP21 capillary column 10 mm, I.D. 0.32 mm, film thickness 0.25 mm (SGE Pty Ltd., Australia) and a flame ionisation detector (FID). Briefly, 0.1 g of individual faecal samples were weighed into 1.5 mL centrifuge tubes into which 1 mL of water containing 2% orthophosphoric acid was dispensed. A 20-μL of internal standard (1 mmol/L of 4-methyl valerate) was added to each sample which was then briefly vortexed and incubated for 30 min at room temperature. The samples were centrifuged at 12,000 r/min for 10 min. The supernatants were transferred with a disposable glass Pasteur pipette to their corresponding 6 mL scintillation vials and 2 mL of diethyl ether was added into each sample which was then briefly vortexed. The upper layer of diethyl ether was transferred into corresponding gas chromatography vials and run for SCFAs analysis. A programmed temperature ramp (50-220 °C) was used. Helium gas was utilised as a carrier at a flow rate of 3 mL/min in the column and the inlet split ratio was set at 20:1. The identification and quantitation of SCFAs were achieved by comparing the retention times and a peak area of unknown samples to that of commercial lipid standard (4-methylvaleric acid) as an internal control.

### *Salmonella* Typhimurium enumeration in organs

At week 30 of flock age, the laying chickens were humanely euthanised by cervical dislocation and tissue pieces of various organs (spleen, liver, ovary, infundibulum/magnum, shell gland, jejunum and cecum) were aseptically collected into 1.5 mL Safe-Lock Eppendorf tubes containing stainless steel beads 0.5-2.0 mm and 500 μL PBS. After weighing, tissues were homogenized using a bullet blender (Next Advance, USA) on full speed for 5-10 min. From the original tissue homogenates or serially diluted samples (cecum), 100 μL was plated onto XLD agar and incubated overnight at 37 °C. *Salmonella* load was expressed as log_10_ CFU/g of tissue. A 100-μL from the original homogenates was also enriched into 900 μL buffered peptone water (BPW) and processed for *Salmonella* isolation through the enrichment method [40]. Putative *Salmonella* colonies on XLD were streaked on Brilliance *Salmonella* agar (BSA; Oxoid, Australia) plates and incubated overnight at 37 °C for confirmation. Incubated plates were read as positive (scored as 1) or negative (scored as 0) for *Salmonella* based on the colony characteristics.

### Faecal DNA extraction and 16S rRNA sequencing

Faecal DNA was extracted following the protocol of QIAamp FAST DNA Mini Kit with the inclusion of homogenisation step with glass beads (acid-washed ≤106 μm and 425-600 μm; Sigma Aldrich, Australia). The DNA quality was tested using a Nanodrop-1000 and the samples (*n* = 378) were submitted to the Ramaciotti Centre for Genomics (University of New South Wales, Australia) for 16S rRNA sequencing and generation of operational taxonomic units (OTUs) table. For generating 2 × 300 bp paired-end reads in Illumina, V3-V4 region specific primer pair (341F: 5′-CCTACGGGNGGCWGCAG-3′; 805R: 5′-GACTACHVGGGTATCTAATCC-3′) was used.

### 16S rRNA library preparation and Illumina sequencing

The library was prepared using barcoding PCR in a 25-μL reaction volume that contained 12.5 μL KAPA HiFi HotStart Readymix (Kapa Biosystems), 1 μL of each the primers, 1 μL DNA template and 10.5 μL PCR grade water. The thermal cycling conditions in SimpliAmp Thermal Cycler (Applied Biosystems) were: initial denaturation at 95 °C for 3 min, 35 cycles of denaturation at 95 °C for 30 s, annealing at 55 °C for 30 s and elongation at 72 °C for 30 s, ending with a final elongation at 72 °C for 5 min. The PCR products were normalised and pooled using SequalPrep™ Normalization Plate Kit (ThermoFisher Scientific, Australia) according to the manufacturer’s instructions. The library was purified using Axygen AxyPrep Mag PCR Clean-Up Kit (Fisher biotec, Australia) as per the manufacturer’s instructions. Concentration and quality of the pooled library were checked with Qubit and the library size on an Agilent 2200 TapeStation instrument. The Agencourt AMPure XP Bead Clean-up kit was used on the pool to reduce/remove the presence of primer dimers. The library pool was sequenced on Illumina MiSeq using a MiSeq Reagent Kit v3 with a 2 × 300 bp run format, using default run parameters including adaptor trimming. For these runs, custom primers were added to the reagent cartridge for Read1, Index and Read2.

### Microbial community data analysis for generation of OTU table

Reads were processed with mothur (v1.39.5) [43] according to the MiSeq protocol [44]. Briefly, the reads were quality filtered and assigned to their respective samples. Samples were trimmed and only those with a length between 405 and 495 bp were retained. Samples with homopolymers longer than 8 bp were removed. Chimeric sequences were removed using the chimera.vsearch script in mothur [45]. The sequences were aligned and classified against the SILVA reference alignment (v132) [45] and lineages not targeted by the primer pair (i.e. archaea, chloroplast, eukaryote, mitochondria and unknown) were removed. Sequences were grouped into OTUs based on 97% similarity using the OptiClust algorithm [46] and subsampled based on the sample with the lowest number of sequences, i.e. 25,556 sequences. Sequencing error was assessed using the ZymoBIOMICS Microbial Community Standard as control in each sequencing run. Interactive OTU plots were created with Krona [47] from the subsampled data. OTU richness plot was generated with the mothur_krona_XML.py script [48]. Diversity plots were generated by using the OTUsamples2krona.sh script [49] by providing a reformatted mothur biom file.

### Statistical analysis

The *Salmonella* Typhimurium load data in faeces (log_10_ mMPN) and in organs (mean percent value) were analysed in Statview software (Version 5.0.1.0) by taking treatment and sampling time-point as main effects. Level of significance was determined by Fisher’s protected least significant difference (PLSD) at *P* < 0.05. For microbial community profiling, the OTU table was analysed in Calypso software [50] using one- and two-way ANOVA, redundancy analysis (RDA+), regression and diversity analyses. To remove the non-independence of relative microbial abundance, the data were transformed using the total sum normalisation (TSS) method [50, 51]. TSS normalises count data by dividing feature read counts by the total number of reads in each sample for obtaining relative abundance [50]. RDA is used to calculate complex association between microbial community composition and explanatory variables. In Calypso, false discovery rate (FDR) of < 0.05 was used for level of significance between the treatment groups.

## Results

### 16S rRNA data and its quality

The sequenced reads quality was as per Q30 standard and the average reads generated per sample were enough for genome alignment and the generation of OTU table for downstream analysis. The rarefaction analysis showed that the sequenced data covered well the diversity of the studied microbiota (Additional file 1: Figure S1). Overall, at phylum level, the microbial communities were clustered into Actinobacteria, Bacteroidetes, Cyanobacteria, Deferribacteres, Firmicutes, Proteobacteria, Synergistetes, Tenericutes and Verrucomicrobia (Additional file 2: Figure S2).

### Gut microbiota abundance and diversity are affected by *Salmonella* Typhimurium challenge

**Fig. 1:**
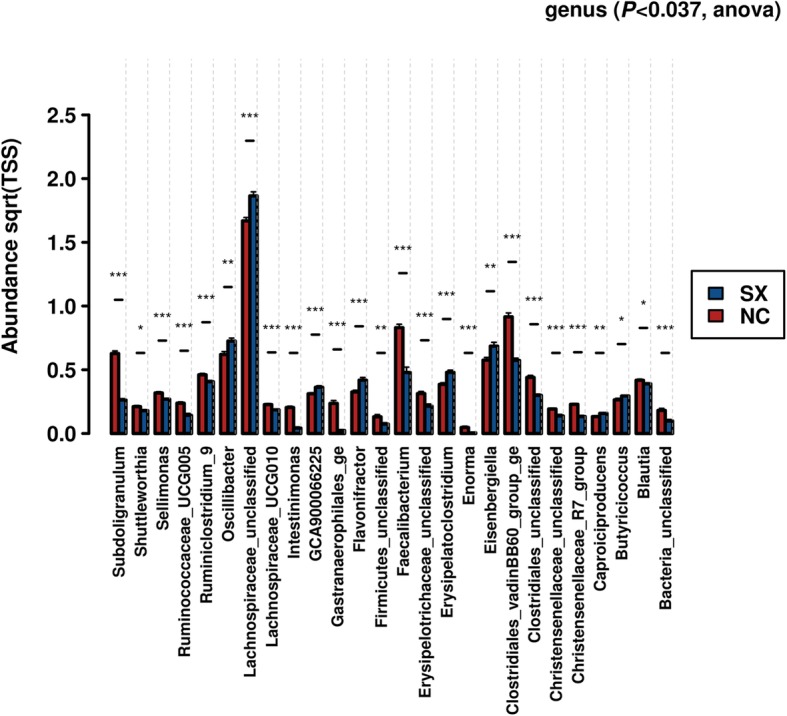
Effect of *Salmonella* Typhimurium challenge on the abundance of gut microbial communities. Compared with the negative control (NC), the abundance levels of different genera were significantly different in the *Salmonella* Typhimurium challenged (SX) group. Only significant genera (* indicates the level of significance) between the two treatment groups have been depicted here. In the Calypso software, *P* < 0.037 was equivalent to FDR < 0.05

To understand the effects of *Salmonella* Typhimurium on gut microbiota diversity and the abundance levels of different genera, the faecal microbiota data of the challenged laying chickens were analysed against the negative control group. Compared with the negative control, *Salmonella* challenge significantly (FDR < 0.05) reduced the abundance of various bacterial genera that included *Subdoligranulum*, *Shuttleworthia*, *Sellimonas*, Ruminiclostridium_9, *Intestinimonas*, Gastranaerophilales_ge, *Faecalibacterium*, *Enorma* and *Blautia* (Fig. 1). The abundance levels of *Oscillibacter*, GCA900066225, *Flavonifractor*, *Erysipelatoclostridium*, *Eisenbergiella*, *Caproiciproducens* and *Butyricicoccus* were significantly increased in the *Salmonella* Typhimurium challenged group compared with the negative control group. The abundance of these genera was also visualised in individual samples of the same chickens obtained at different sampling time-points (Additional file 3; Figure S3). The abundance of *Bacteroides* increased after week 8 post-challenge both in the negative control and *Salmonella* challenged groups.

**Fig. 2:**
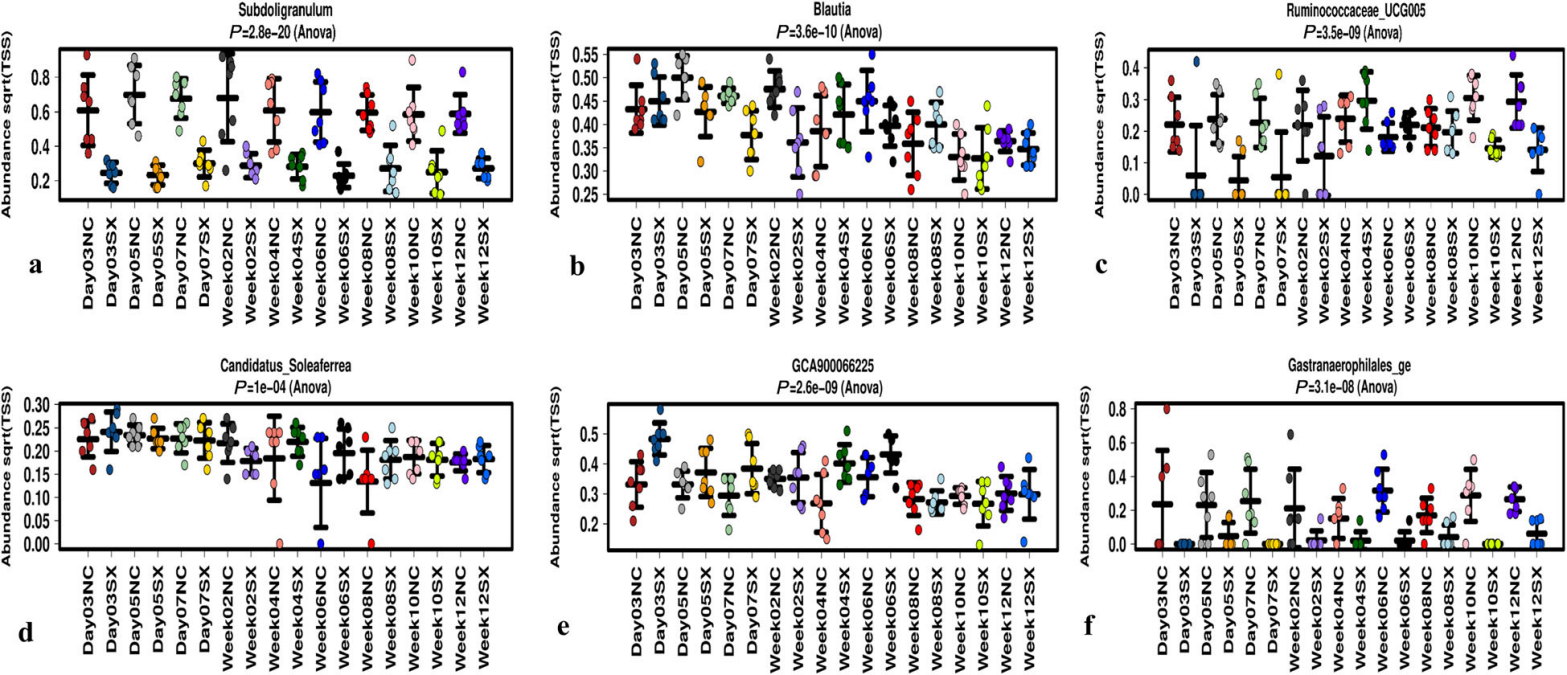
The microbial abundance of individual genera affected by *Salmonella* Typhimurium challenge. The microbial abundance at the genera level of the negative control (NC) group was compared with the *Salmonella* Typhimurium challenged (SX) group. The data from the samples collected at nine different sampling time-points (days 3, 5, 7 and weeks 2, 4, 6, 8, 10 and 12) were visualised between the NC and SX groups. Abundance levels of (**a**) Subdoligranulum; **b** Blautia; **c** Ruminococcaceae_UCG005; **d** Candidatus_Soleaferrea; **e** GCA900066225 and **f** Gastranaerophilales_ge at different time-points between the NC and SX groups

**Fig. 3:**
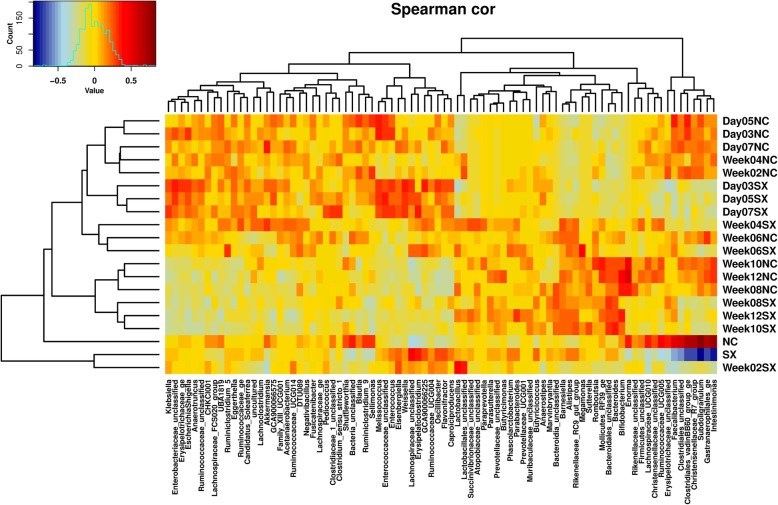
Heatmap showing the abundance of individual microbial communities affected by *Salmonella* Typhimurium challenge and sampling time-points. The abundance levels of different microbial genera of the negative control group (NC) were clearly separated from the *Salmonella* challenged (SX) group. Data obtained from the faecal samples collected on days 3, 5, 7 and weeks 2, 4, 6, 8, 10, and 12 post-challenge were visualised

**Fig. 4:**
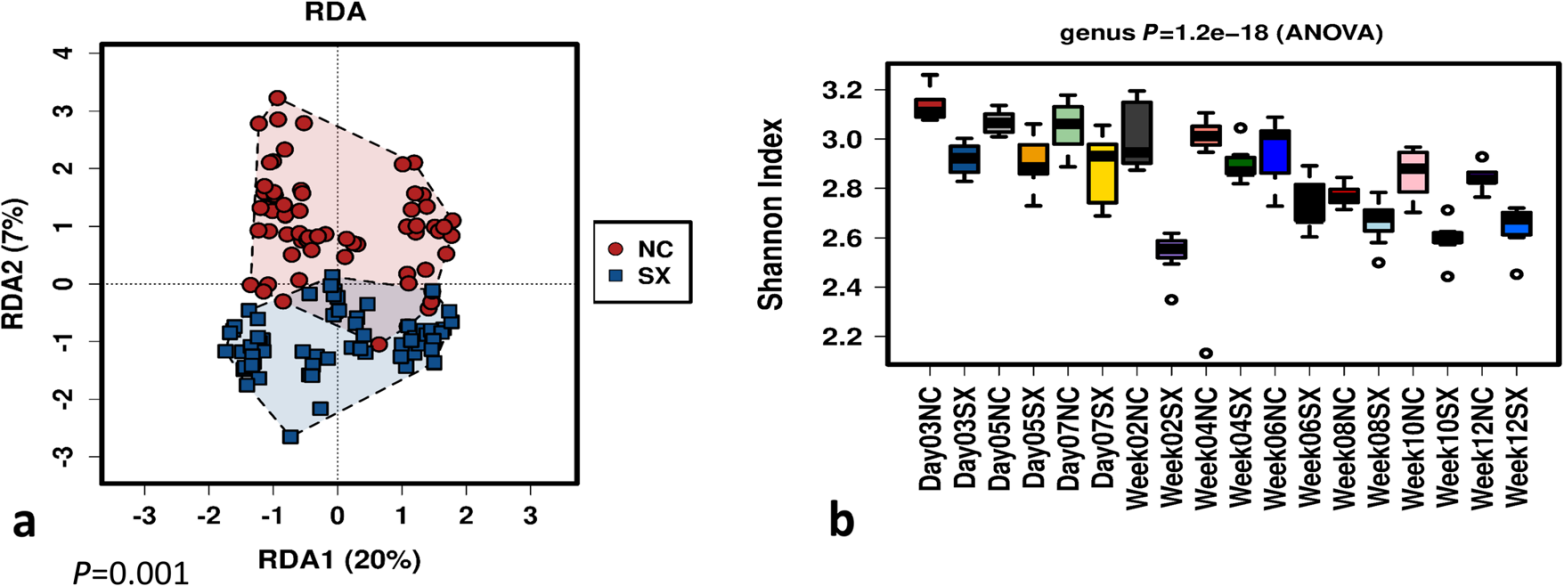
Microbial community composition and diversity affected by *Salmonella* Typhimurium challenge. **a** Microbial community composition between the negative control (NC) and *Salmonella* Typhimurium challenged (SX) groups. **b** Microbial diversity between the NC and SX at different time-points (days 3, 5, 7 and weeks 2, 4, 6, 8, 10 and 12) post-challenge

A significant (FDR < 0.05) effect of sampling time-point was observed on the abundance of multiple genera between the negative control and *Salmonella* Typhimurium challenged groups (Fig. 2; Additional file 4: Figure S4). The abundance levels of different genera varied differently with sampling time-points. Genera such as *Subdoligranulum*, Gastranaerophilales_ge, *Intestinimonas*, Ruminococcaceae_UCG005 and *Sellimonas* were consistently lower in abundance in the *Salmonella* Typhimurium challenged group at all sampling time-points. A correlation heatmap was used to understand the effects of the sampling time-points and *Salmonella* Typhimurium challenge on the abundance of individual genera of gut microbial communities. A clear pattern of representation of individual microbial communities at different time-points both in the negative control and *Salmonella* Typhimurium challenged groups shows that *Salmonella* challenge affected the abundance of multiple microbial genera (Fig. 3). Measured by redundancy analysis (RDA+), there was a significant (*P* < 0.05) effect of *Salmonella* challenge on the microbial community composition (Fig. 4a). The microbial alpha diversity (measured as Shannon index at genera level) was significantly different between the negative control (NC) and the *Salmonella* Typhimurium challenged (SX) group (Fig. 4b). The microbial diversity was significantly lower in the SX group across all the sampling time-points. Around week 4 post-challenge, two out of seven chickens were consistently negative for *Salmonella* Typhimurium. The gut microbiota analysis of the two *Salmonella* negative chickens showed a significantly higher abundance of *Faecalibacterium*, Erysipelotrichaceae_unclassified, Rikenellaceae_RC9_gut_group and *Intestinimonas* (Additional file 5: Figure S5).

To understand the effects of the probiotic on gut microbiota in the presence of *Salmonella* Typhimurium, the abundance of microbial genera was compared between the CPC and CPX and between the IPC and IPX groups. Compared with the CPC, *Salmonella* Typhimurium challenge significantly decreased the abundance levels of *Acetanaerobacterium*, *Akkermansia*, *Anaerostipes*, *Bacteroides*, *Blautia*, *Eggerthella*, *Eisenbergiella*, *Enterococcus*, *EscherichiaShigella*, *Faecalibacterium*, *Lactobacillus*, *Melissococcus*, *Oscillibacter*, *Pediococcus*, Ruminiclostridium_9, Ruminococcaceae_UCG014, *Sellimonas*, *Subdoligranulum* and *Weissella*, while increasing the abundance levels of *Alistipes*, *Barnesiella*, *Bifidobacterium*, *Butyricimonas*, *Enorma*, *Intestinimonas*, *Megamonas*, *Parabacteroides*, *Paraprevotella*, *Parasutterella*, *Phascolarctobacterium* and *Sutterella* (Additional file 6: Figure S6).

The microbial community composition of the CPC was significantly separated from the CPX group (Additional file 7: Figure S7a). The microbial diversity was significantly lower in the CPC compared with the CPX across all the sampling time-points (Additional file 7: Figure S7b). The abundance levels of microbial genera in the IPC and IPX treatment groups were comparable to the CPC and CPX treatment groups, but there were fewer genera significantly affected between the two treatment groups of IPC and IPX (Additional file 8: Figure S8). The microbial community composition of the IPX group was clearly separated from the IPC group and diversity of the IPX treatment group was significantly higher than the IPC in some of the sampling time-points (Additional file 9: Figure S9a, b).

To determine the effects of probiotic supplementation on microbial abundance and diversity, data were analysed and compared between the negative control and the probiotic supplemented control groups (excluding *Salmonella* Typhimurium challenge). Compared to the negative control, the continuous supplementation of the probiotic decreased the diversity of microbiota (Additional file 10: Figure S10a) and the abundance of *Eisenbergiella*, *EscherichiaShigella*, *Blautia*, *Flavonifractor* and *Subdoligranulum* (Additional file 10: Figure S10b). Compared with the negative control, the intermittent supplementation of probiotic decreased the diversity of microbiota (Additional file 11: Figure S11a) and the abundance of microbial genera, such as *Faecalibacterium*, *EscherichiaShigella*, *Blautia*, *Sellimonas* and *Subdoligranulum* (Additional file 11: Figure 11b).

### Gut microbiota displaced by *Salmonella* Typhimurium was restored by *Bacillus* based probiotic supplementation

**Fig. 5:**
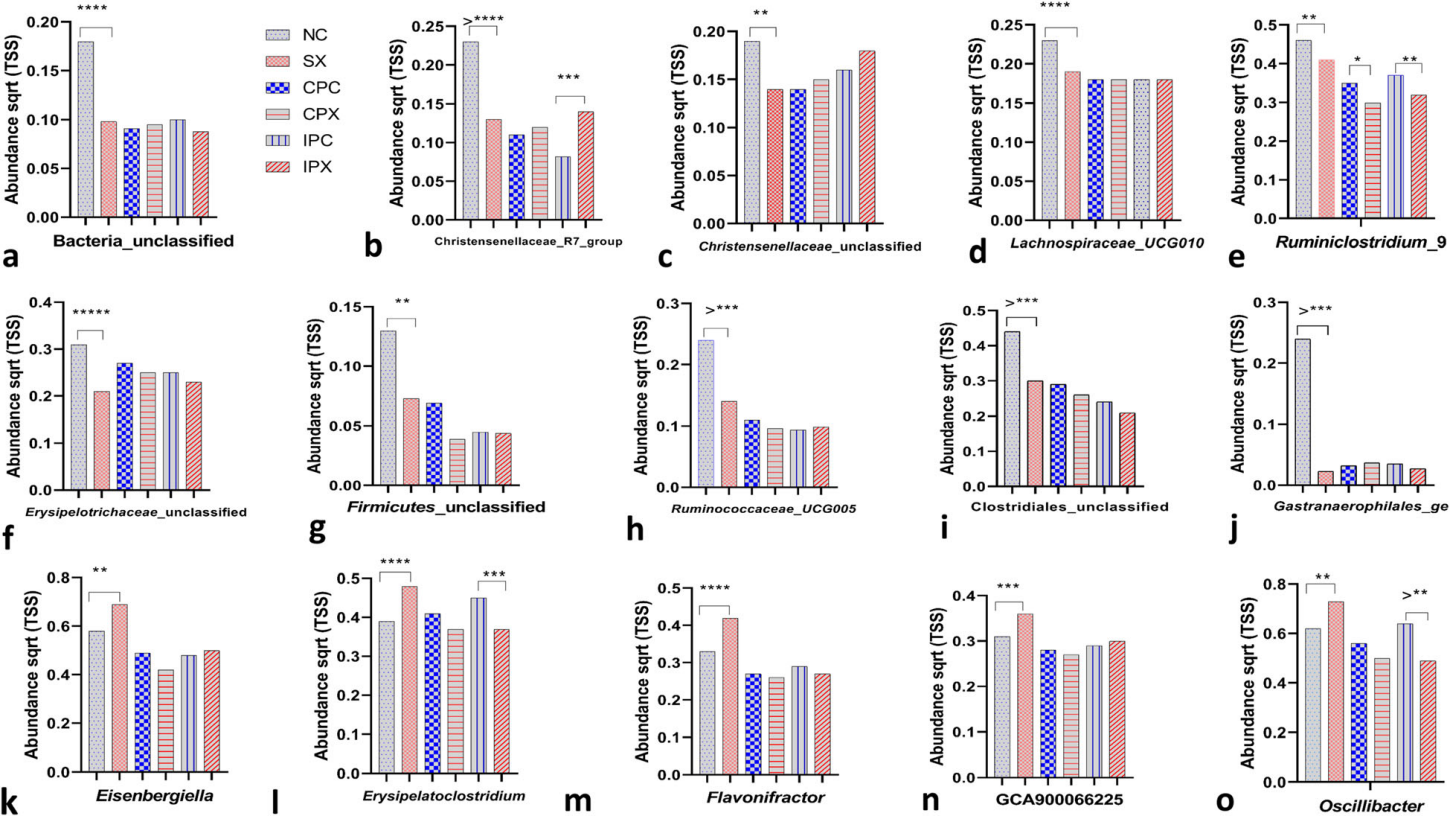
*Bacillus* based probiotic can restore the abundance of microbial communities (genera level analysis) that are displaced by *Salmonella* Typhimurium challenge. For direct group comparisons, the level of significance (if any) has shown between the negative control (NC) and *Salmonella* challenged (SX), the continuously supplemented probiotic control (CPC) and continuous supplemented probiotic and *Salmonella* challenged (CPX) and the intermittent supplemented probiotic control (IPC) and intermittent supplemented probiotic and *Salmonella* challenged (IPX) groups. The number of asterisks shows the level of significance (Tukey’s *P* value). Abundance levels of **a** Bacteria_unclassified; **b** Christensenellaceae_R7_group; **c** Christensenellaceae_unclassified; **d** Lachnospiraceae_UCG010; **e** Ruminiclostridium_9; **f** Erysipelotrichaceae_unclassified; **g** Firmicutes_unclassified; **h** Ruminococcaceae_UCG005; **i** Clostridiales_unclassified; **j** Gastranaerophilales_ge; **k***Eisenbergiella*; **l***Erysipelatoclostridium*; **m***Flavonifractor*; **n** GCA900066225 and **o***Oscillibacter* between the treatment groups NC and SX, CPC and CPX and IPC and IPX

To understand the effects of the *Bacillus* based probiotic in restoring the gut microbial community abundance, we analysed the data obtained from the chickens continuously or intermittently fed with probiotic supplement and challenged with *Salmonella* Typhimurium or left as probiotic controls. The data were analysed against each respective treatment groups. The abundance levels of microbial genera that were significantly decreased or increased by the *Salmonella* Typhimurium challenge (SX) compared with the negative control (NC) group, were assessed for the effects of the probiotic. Probiotic supplementation restored (FDR > 0.05) the abundance levels of microbial genera, such as Bacteria_unclassified, Christensenellaceae_R7_group, Christensenellaceae_unclassifed, Lachnospiraceae_UCG010, Ruminiclostridium_9, Erysipelotrichaceae_unclassified, Firmicutes_unclassified, Ruminococcaceae_UCG005, Clostridiales_unclassified and Gastranaerophilales_ge (Fig. 5 a-j). Compared with the negative control, *Salmonella* challenge significantly increased the abundance of *Eisenbergiella*, *Erysipelatoclostridium*, *Flavonifractor*, GCA900066225 and *Oscillibacter* (Fig. 5 k-o). When the effects of the continuously and intermittently supplemented *Bacillus* based probiotic on the restoration of the abundance of these microbial communities were assessed, the data showed that the continuously and intermittently supplemented probiotic restored microbiota with clearer effects observed for the continuously supplemented probiotic (Fig. 5 a-o).

### Microbial abundance affected by *Salmonella* Typhimurium was different in the presence of probiotic

**Fig. 6:**
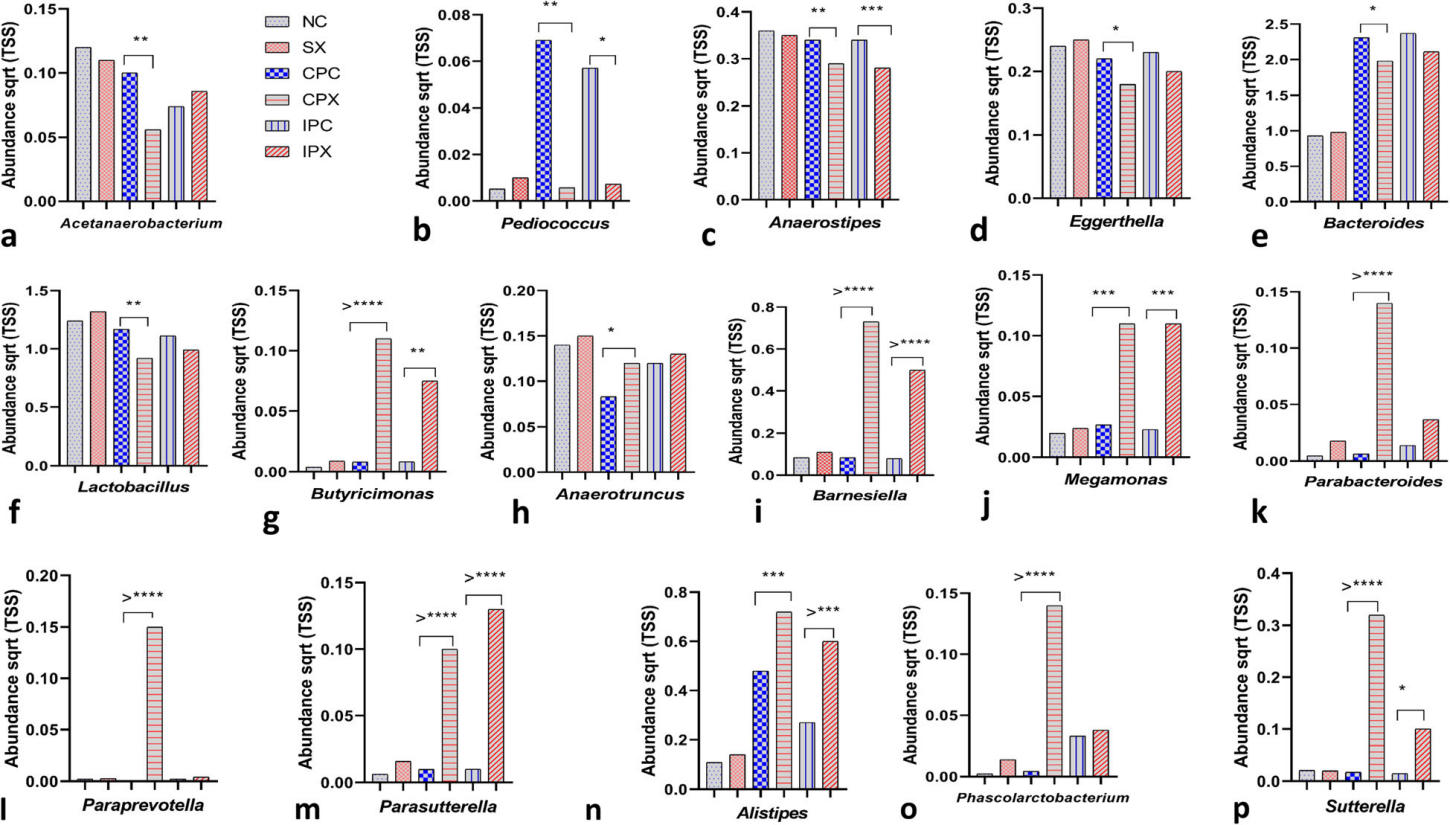
The abundance of microbial genera affected by *Salmonella* Typhimurium in the presence of *Bacillus* based probiotic. For direct group comparisons, the level of significance (if any) has shown between the negative control (NC) and *Salmonella* challenged (SX), the continuous supplemented probiotic control (CPC) and continuously supplemented probiotic and *Salmonella* challenged (CPX) and the intermittent supplemented probiotic control (IPC) and intermittent supplemented probiotic and *Salmonella* challenged (IPX) groups. The number of asterisks shows the level of significance (Tukey’s *P* value). Abundance levels of **a***Acetanaerobacterium*; **b***Pediococcus*; **c***Anaerostipes*; **d***Eggerthella*; **e***Bacteroides*; **f***Lactobacillus*; **g***Butyricimonas*; **h***Anaerotruncus*; **i***Barnesiella*; **j***Megamonas*; **k***Parabacteroides*; **l***Paraprevotella*; **m***Parasutterella*; **n***Alistipes*; **o***Phascolarctobacterium* and **p***Sutterella* between the treatment groups NC and SX, CPC and CPX and IPC and IPX

The effect of the *Bacillus* based probiotic on the abundance of microbial communities at genera level in the presence and absence of *Salmonella* Typhimurium challenge was also assessed. Compared with the probiotic supplemented control groups, *Salmonella* challenge significantly reduced the abundance of *Acetanaerobacterium*, *Pediococcus*, *Anaerostipes*, *Eggerthella*, *Bacteroides* and *Lactobacillus* in the probiotic supplemented and *Salmonella* Typhimurium challenged groups (Fig. 6a-f). This effect was highly significant for the continuously supplemented probiotic compared with the intermittently supplemented probiotic group (Fig. 6a-f). Interestingly, the abundance of *Butyricimonas*, *Anaerotruncus*, *Barnesiella*, *Megamonas*, *Parabacteroides*, *Paraprevotella*, *Parasutterella*, *Alistipes*, *Phascolarctobacterium* and *Sutterella* was significantly higher in the probiotic supplemented and *Salmonella* challenged groups compared with the probiotic supplemented control groups (Fig. 6g-p). The abundance of these microbial communities was not significantly different between the negative control and *Salmonella* challenged groups (Fig. 6a-p).

### Gut microbiota drives *Salmonella* Typhimurium load

To understand the interaction of *Salmonella* Typhimurium load with the gut microbiota at individual genera level, a regression analysis was performed on the log_10_ mMPN values of individual birds against each of the genera in the *Salmonella* challenged (SX) group. The load of *Salmonella* Typhimurium in the gut significantly (*P* < 0.05) affected the abundance of different microbial genera. The abundance of 30 microbial genera showed a significant weak negative correlation with the *Salmonella* Typhimurium load in the gut (Table 1). These genera included important gut resident microbiota members such as *Lactobacillus*, *Megamonas*, *Enorma*, *Barnesiella*, *Butyricimonas*, *Faecalibacterium*, *Intestinimonas* and *Parabacteroides*. The abundance of 24 microbial genera showed a significant weak positive correlation with the *Salmonella* Typhimurium load in the gut (Table 1). These microbial communities included genera such as *Acetanaerobacterium*, *Akkermansia*, *Anaerostipes*, *Blautia*, *Eggerthella*, *Pediococcus* and *EscherichiaShigella*.

**Table 1:** Correlation of *Salmonella* Typhimurium load with abundance of microbial genera in faeces

Microbe	R value	*P* value	Microbe	R value	*P* value
*Alistipes*	−0.341	1.60E-06	Prevotellaceae_UCG001	−0.323	5.70E-06
Atopobiaceae_unclassified	−0.442	1.80E-10	Prevotellaceae_unclassified	−0.297	3.30E-05
Bacteroidales_unclassified	−0.279	1.00E-04	Rikenellaceae_RC9-gut-group	−0.258	3.30E-04
*Bifidobacterium*	−0.531	3.70E-10	*Acetanaerobacterium*	0.181	0.013
*Barnesiella*	−0.433	5.00E-10	*Akkermansia*	0.212	0.0034
*Butyricimonas*	−0.233	1.30E-03	*Anaerostipes*	0.153	0.036
Christensenellaceae_R7_group	−0.146	4.40E-02	*Anaerotruncus*	0.179	0.014
Clostridiales_vadinB860_group_ge	−0.346	1.10E-06	*Blautia*	0.258	0.00033
*Enorma*	−0.403	9.10E-09	*Caproiciproducens*	0.192	0.0082
*Faecalibacterium*	−0.213	3.30E-03	Clostridiaceae_1_unclassified	0.299	2.90E-05
Family_XIII_UCG001	−0.318	8.10E-06	Clostridium_sensu_stricto_1	0.25	0.00051
*Intestinimonas*	−0.301	2.50E-05	*Eggerthella*	0.39	2.90E-08
*Lactobacillus*	−0.384	4.80E-08	*Eisenbergiella*	0.416	2.70E-09
*Megamonas*	−0.309	2.60E-05	Enterococcaceae_unclassified	0.397	1.60E-08
*Negativibacillus*	−0.168	2.10E-02	*Erysipelatoclostridium*	0.391	2.70E-08
*Parabacteroides*	−0.301	2.60E-05	Erysipelotrichaceae_ge	0.29	5.00E-05
*Paraprevotella*	−0.242	7.90E-04	*EscherichiaShigella*	0.542	8.90E-16
*Parasutterella*	−0.304	2.10E-05	*Flavonifractor*	0.268	1.90E-04
*Phascolarctobacterium*	−0.194	7.50E-03	*Fusicatenibacter*	0.21	3.70E-03
*Romboutsia*	−0.304	2.10E-05	GCA900066575	0.422	1.50E-09
*Sutterella*	−0.377	9.20E-08	Lachnospiraceae_unclassified	0.267	0.00021
Succinivibrionaceae_unclassified	−0.26	3.00E-04	*Melissococcus*	0.252	4.60E-04
Ruminococcaceae_UCG005	−0.355	5.50E-07	*Pediococcus*	0.22	2.40E-03
Rikenellaceae_unclassified	−0.318	8.40E-06	*Ruminiclostridium_5*	0.279	1.00E-04
Bacteroidia_unclassified	−0.186	1.00E-02	Ruminococcaceae_unclassified	0.146	4.60E-02
Mollicutes_RF39_ge	−0.281	8.90E-05	*Sellimonas*	0.333	2.90E-06
Muribaculaceae_unclassified	−0.309	1.80E-05	*Weissella*	0.352	6.70E-07

The faecal load of *Salmonella* Typhimurium (in log_10_ mMPN) was regressed against the abundance of individual genera of microbiota. Minus (−) sign shows negative correlation

### Short chain fatty acids quantification from faeces

**Fig. 7:**
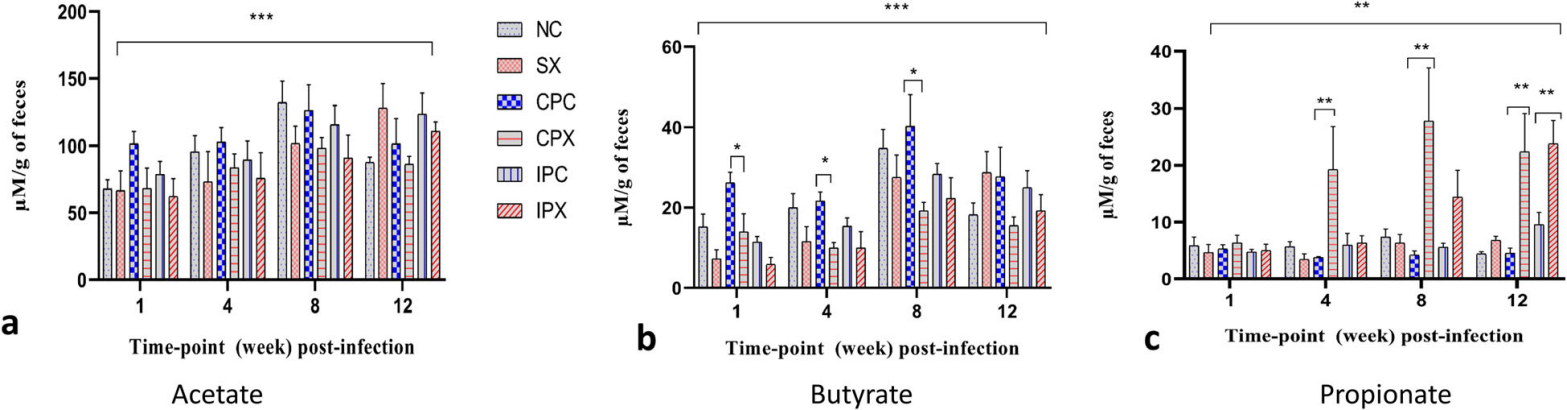
Short-chain fatty acids levels in the faeces of chickens fed with probiotic and challenged with *Salmonella* Typhimurium. The respective treatment groups were compared with each other at each sampling time-point of *Salmonella* Typhimurium post-challenge. **a** Acetate, **b** butyrate and **c** propionate levels in faeces at different sampling time-points (weeks 1, 4, 8 and 12 post-challenge). Bar (with asterisks) across the individual graph shows a significant effect of treatment group on short-chain fatty acids production. NC is negative control; SX is *Salmonella* challenge; CPX is continuous probiotic supplemented and *Salmonella* challenge; CPC is continuous probiotic supplemented control; IPX is intermittent probiotic supplemented and *Salmonella* challenge; IPC is intermittent probiotic control

The levels of acetate, butyrate and propionate were significantly (*P* < 0.05) affected over time following *Salmonella* Typhimurium infection (Fig. 7). Among the treatment groups, the levels of acetate and butyrate were significantly higher in the continuously supplemented probiotic control (CPC) compared with the continuously supplemented probiotic and *Salmonella* Typhimurium challenged (CPX) group. However, within each treatment group, at each sampling time-point, there was no significant (*P* > 0.05) difference in the acetate content of the faeces (Fig. 7a). Within each treatment group, the level of butyrate in the faeces was significantly higher in the CPC and intermittent supplemented probiotic control (IPC) groups compared with the CPX and the intermittent supplemented probiotic and *Salmonella* Typhimurium challenged (IPX) groups. Within each treatment group, the level of butyrate in the faeces at week 1, 4 and 8 post-challenge was significantly higher in the CPC compared with the IPX group (Fig. 7b). The propionate level was significantly affected by the sampling time-point post *Salmonella* Typhimurium challenge but was not consistent with the levels of acetate and butyrate (Fig. 7c). Within each treatment group, the level of propionate in the faeces was significantly lower in the CPC and IPC compared with the CPX and IPX groups.

### Effects of probiotic supplementation on *Salmonella* Typhimurium load in faeces and organs

**Fig. 8:**
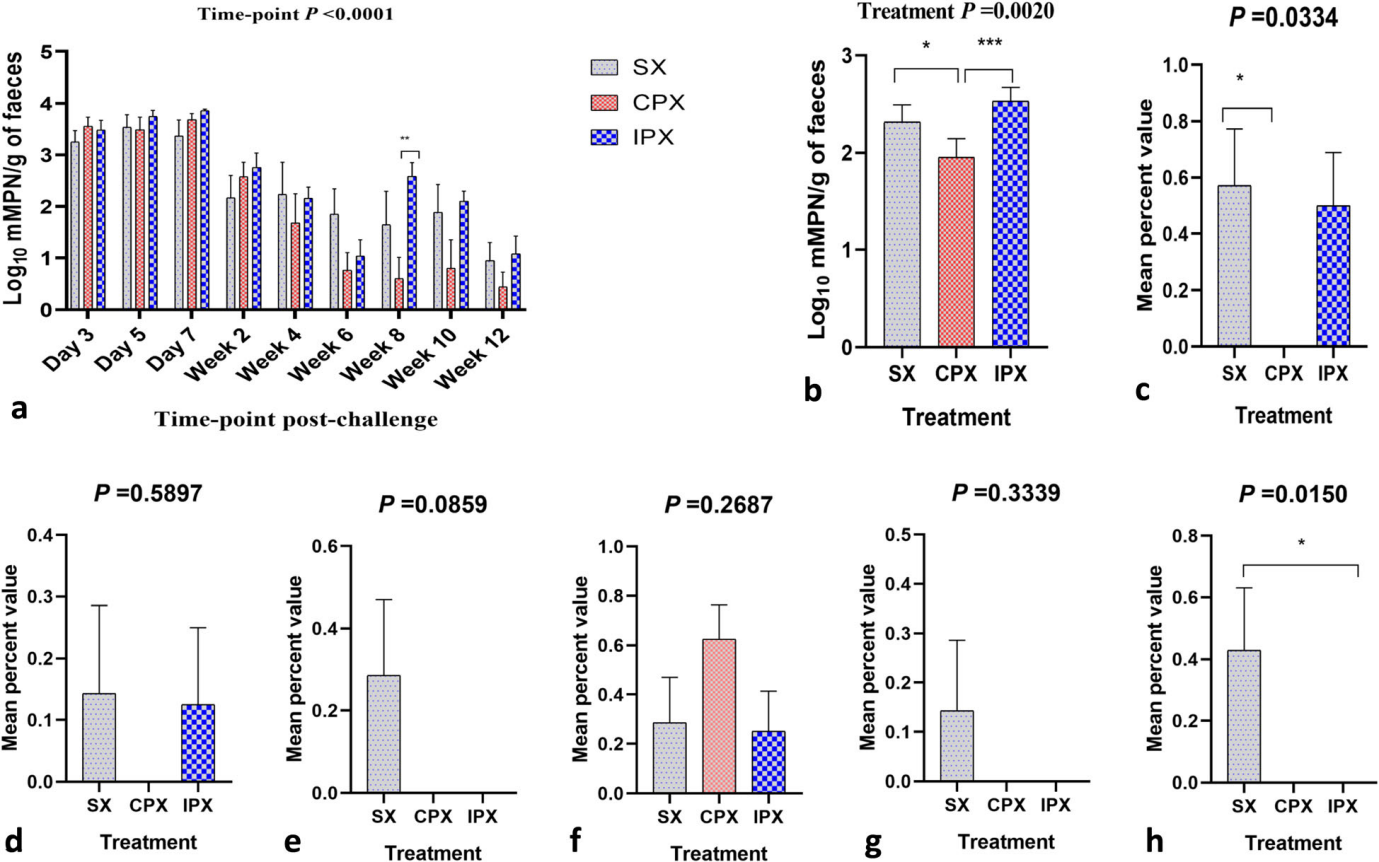
Load of *Salmonella* Typhimurium in faeces affected by sampling time-points and mean percent value of *Salmonella* Typhimurium in organs at week 12 post-challenge. **a***Salmonella* load in log_10_ mMPN per gram of faeces at different sampling time-points in three treatment groups (SX- *Salmonella* Typhimurium challenged; CPX- *Salmonella* Typhimurium challenged and continuously supplemented feed with probiotic; IPX- *Salmonella* Typhimurium challenged and continuously supplemented feed with probiotic). **b***Salmonella* load in log_10_ mMPN per gram of faeces affected by treatment groups. Mean percent value of *Salmonella* Typhimurium in **c** cecum; **d** jejunum; **e** liver; **f** spleen; **g** magnum/infundibulum and **h** shell gland. For *Salmonella* load in the organs, small pieces of the tissues with known weight were homogenised in 500 μL PBS and a 100-μL was plated on XLD media. From the same tissue homogenates, a 100-μL was enriched in BPW followed by RVS and streaked on XLD/BSA media. The XLD/BSA plates were read as positive (1) and negative (0). The data were analysed in Statview software for getting mean percent values that represent the load of *Salmonella* Typhimurium in organs

To understand the effects of gut microbiota modulation through the probiotic on *Salmonella* Typhimurium shedding levels in faeces, an mMPN method (log_10_) was performed. Faeces from the negative and probiotic control groups were negative for *Salmonella*. Irrespective of the probiotic supplementation, some chickens from the *Salmonella* challenged groups turned negative for *Salmonella* Typhimurium load in faeces around week 4 post-challenge. However, not all of these chickens were consistently negative for faecal load of *Salmonella* at different sampling time-points. A significant effect of time-point and treatment was observed on the shedding level of *Salmonella* Typhimurium in the faeces (Fig. 8a, b). Within each sampling time-point, the continuously supplemented probiotic and *Salmonella* Typhimurium challenged group (CPX) showed a significantly lower bacterial load compared with the intermittent supplemented probiotic and *Salmonella* Typhimurium challenged group (IPX) at week 8 post-challenge (Fig. 8a). Overall, the load of *Salmonella* Typhimurium was significantly lower in the CPX compared with the *Salmonella* challenged (SX) and IPX groups (Fig. 8b).

The load of *Salmonella* Typhimurium in organs was determined at the point of termination of the experiment (week 30 of flock age). *Salmonella* was not recovered from various organs collected from the negative and probiotic control groups. For the *Salmonella* Typhimurium challenged groups, organ homogenates directly plated on XLD and BSA media were negative; however, some samples turned positive when an enrichment method was followed. Therefore, the load of *Salmonella* Typhimurium in organs was expressed as mean percent value per treatment group. The mean percent value of *Salmonella* Typhimurium for caecum was significantly lower in the CPX compared with the SX group (Fig. 8c) The mean percent value of *Salmonella* Typhimurium for shell gland was significantly lower in the CPX and IPX compared with the SX group (Fig. 8h). *Salmonella* Typhimurium was not recovered from the cecum, jejunum, liver, magnum/infundibulum and shell gland of the CPX group (Fig. 8c, d, e, g, h). Similarly, *Salmonella* Typhimurium was not recovered from the liver, magnum/infundibulum and shell gland of the IPX group (Fig. 8e, g, h). No *Salmonella* was isolated from the internal contents of the eggs. Shell wash samples positive for *Salmonella* through the enrichment method showed no measurable load by the mMPN method.

## Discussion

The main objectives of this study were to understand the dynamics of the gut microbiota in *Salmonella* Typhimurium infected laying chickens, and to study the effects of continuous and intermittent feeding of probiotic on *Salmonella* Typhimurium shedding. A balanced gut microbiota can resist pathogen colonisation and subsequent clearance from the gut [52]. In this study, we reared *Salmonella* spp. free laying chickens to understand the true effects of this pathogen on gut microbiota displacement as other species of *Salmonella,* if already colonised in gut, can significantly influence the shedding of *Salmonella* Typhimurium. The results showed that both the *Salmonella* Typhimurium and the *Bacillus* based probiotic significantly affected the composition and diversity of the gut microbial communities. The data also showed that continuous supplementation of the *Bacillus* based probiotic reduced the load of *Salmonella* Typhimurium in the faeces (overall) and in organs tested at the end of the experiment. The decrease in abundance levels of *Eisenbergiella*, *EscherichiaShigella*, *Blautia*, *Flavonifractor* and *Subdoligranulum* by the probiotic supplementation shows that the *Bacillus* based probiotic has the potential to affect gut microbial abundance. Of the reduced microbial genera, *Escherichia* and *Shigella* have the potential to cause infection in certain conditions, while other genera such as *Blautia*, *Flavonifractor* and *Subdoligranulum* are vital for gut health. The probiotic supplementation also increased the abundance levels of good bacteria, such as *Bacteroides* and *Alistipes*. Further investigation is necessary to determine the effects of the decreased abundance of the above mentioned microbial genera on the host gut.

In chickens, the composition of gut microbiota varies considerably with bird age, with more complex microbiota present in older birds [53]. *Salmonella* Typhimurium induces inflammation of intestinal epithelia [37] and displacement of gut microbiota in laying chicks [54]. However, the long-term effects of *Salmonella* Typhimurium on the gut microbiota in laying chickens have not been investigated. To understand the role of *Salmonella* Typhimurium colonisation on the abundance and diversity of gut microbiota, the microbial communities of the faeces of individual birds collected at nine different time-points post-challenge from the *Salmonella* negative control and *Salmonella* challenged groups were compared. Overall, the *Salmonella* Typhimurium infection reduced the abundance of many bacterial genera including *Blautia*, Bacteria_unclassified, Christensenellaceae_R7_group, *Enorma*, *Faecalibacterium*, Christensenellaceae_unclassifed, Lachnospiraceae_UCG010, Ruminiclostridium_9, *Subdoligranulum* and Firmicutes_unclassified. This shows that not all members of the gut microbiota have the potential to compete with the *Salmonella* Typhimurium. Most of these bacterial genera play a vital role in maintaining gut health through the production of organic acids and vitamins. For example, Christensenellaceae contains bacteria that secrete β-glucosidase, β-galactosidase and α-arabinosidase and therefore help in polysaccharide digestion [55]. As *Salmonella* lacks the enzyme, β1–4 linkage required for polysaccharide fermentation, Christensenellaceae could ferment it. However, *Salmonella* Typhimurium challenge reduces the abundance of Christensenellaceae and Lachnospiraceae [54]. Therefore, *Salmonella* infection could lead to the interruption of the Christensenellaceae based polysaccharide fermentation. Ruminiclostridium_9 and Ruminococcaceae_UCG005 are members of Ruminococcaceae that are common gut microbes involved in the breakdown of complex carbohydrates. A decreased abundance of Erysipelotrichaceae was observed in Crohn’s disease [56]. Some species in Clostridiales degrade a variety of fibre and have been identified as producing propionate, acetate and butyrate. Gastranaerophilales_ge obtains its energy by obligate fermentation resulting in the production of organic acids in the gut. A previous study suggested that *Salmonella* Enteritidis reduced the abundance level of *Faecalibacterium* in the chicken gut [57]. The functions of *Faecalibacterium* in the chicken gut are not well characterised; however, it is one of the most abundant resident gut microbes in a human gut [58]. In the current study, the lower abundance levels of the above mentioned microbial communities show that *Salmonella* Typhimurium establishes its niche in the gut at the expense of displacing these bacterial communities leading to *Salmonella* driven dysbiosis. In the current study, the increased abundance of *Faecalibacterium* in the gut of hens that turned negative for *Salmonella* suggest its potential role to be characterised as a probiotic candidate for gut health.

Compared with the negative control group, *Salmonella* Typhimurium challenge increased the abundance of *Eisenbergiella, Erysipelatoclostridium, Flavonifractor,* GCA900066225 *and Oscillibacter. Eisenbergiella is a* rod-shaped, non-proteolytic, non-motile, anaerobic bacteria in the Lachnospiraceae that produces succinate, lactate, butyrate and acetate during fermentation [59]. *Erysipelatoclostridium* is a part of normal gut microbiota but could become an opportunistic pathogen and has been identified as a gut microbiota biomarker in human patients suffering from Crohn’s disease and *Clostridium difficile* infection [60]. In the current study, the non-significant difference in the abundance levels of *Eisenbergiella*, *Flavonifractor*, GCA900066225, Oscillibacter and *Erysipelatoclostridium* between the continuously supplemented probiotic control and the continuously supplemented probiotic and *Salmonella* Typhimurium challenged groups shows the positive effect of the probiotic on gut microbiota. These results are further supported by the positive correlation of the abundance levels of the above-mentioned genera with *Salmonella* load in the gut.

The regression analysis of the *Salmonella* Typhimurium load (measured as log_10_ mMPN/g of faeces) against the abundance of gut microbial genera showed that more genera were negatively affected by the *Salmonella* Typhimurium infection. This indicates that, as the *Salmonella* Typhimurium load decreased over time, these microbial genera had the potential to restore normal abundance. The negatively correlated genera, such as *Lactobacillus*, *Megamonas*, *Negativibacillus*, *Parabacteroides*, *Paraprevotella*, *Parasutterella*, *Phascolarctobacterium*, *Romboutsia*, *Bifidobacterium*, *Butyricimonas*, *Barnesiella*, *Faecalibacterium* and *Intestinimonas* perform vital functions ranging from vitamin synthesis to organic acid production. *Megamonas* contains a gene cluster that encodes secreted cellobiose phosphotransferase system, endo-glucanases and 6-phospho-beta-glucocidase that potentially degrade non-starch polysaccharides to cellobiose in the chicken gut [61]. *Negativibacillus* belongs to Ruminococcaceae with no known functions. *Parabacteroides* improves host metabolism through the production of succinate and secondary bile acids in the gut as shown in mice [62]; however, its functions in chickens have not been investigated. For propionate production, *Parabacteroides*, *Alistipes* and *Paraprevotella* express cobalamin-binding methylmalonyl-CoA mutase and/or methylmalonyl-CoA epimerase [63]. In Firmicutes, *Phascolarctobacterium, Megamonas and Blautia produce propionate through* epimerase, decarboxylase and methylmalonyl-CoA mutase pathways [63]. *Faecalibacterium*, *Subdoligranulum,* and *Phascolarctobacterium* produce butyrate through acetyl/propionyl-CoA carboxylase pathway. In the current study, the reduced abundance of the useful microbial genera by the *Salmonella* Typhimurium challenge would have affected their normal functions vital for maintaining gut health through fermentation. Moreover, most of these microbial communities were positively influenced when the probiotic was supplemented in the diet. The effects of the probiotic on the abundance at the genera level were clearer in the continuously supplemented, rather than the intermittent supplemented group. For example, the continuously supplemented probiotic restored the abundance of Christensenellaceae_R7_group, *Erysipelatoclostridium* and *Oscillibacter*, while the intermittent supplementation merely improved it compared with the *Salmonella* challenged groups. This shows that the continuous supplementation of the probiotic produced better results.

On the other hand, the increased load of *Salmonella* Typhimurium favoured a large number of microbial communities of the gut microbiota by increasing their abundance. The bacterial genera that were positively correlated with the *Salmonella* Typhimurium load included *Flavonifractor*, *Akkermansia*, *Anaerostipes*, *Blautia*, *Caproiciproducens*, *Eggerthella*, *Eisenbergiella*, *Erysipelatoclostridium*, *Melisococcus*, *Pediococcus*, Ruminiclostridium_5, *Sellimonas*, *Weissella* and some unclassified genera. Although these genera are part of normal gut microbiota, some of them can become opportunistic pathogens causing dysbiosis and subsequent infections. The precise molecular mechanisms underlying how *Salmonella* Typhimurium causes the increased abundance of these genera are not known; however, in this study, we showed that the *Salmonella* driven dysbiosis favours a large number of resident gut microbiota to increase in abundance thereby affecting the abundance of other resident gut microbial community members. Flavonifractor is a member of resident gut microbiota but has been shown to cause infection in an immunocompromised patient [64]. The precise role of *Flavonifractor* in the dysbiosed gut of chickens needs to be investigated.

In the current study, the levels of acetate, butyrate and propionate in faeces were quantified at week 1, 4, 8 and 12 post-challenge to understand the effects of the probiotic treatment in *Salmonella* challenged or non-challenged hens. The higher level of butyrate in response to the supplementation of the probiotic shows that the probiotic treatment increased its production, while the *Salmonella* infection decreased it possibly due the displaced microbial communities. The microbiota produced gut metabolites such as acetate, butyrate and propionate. These metabolites play an important role in gut health ranging from the provision of energy to host enterocytes and regulation of the immune system [65]. The propionate level was higher in *Salmonella* challenged and probiotic supplemented groups compared to the probiotic control groups. It seems that certain organic acid producing genera that increased in abundance in response to *Salmonella* infection may have produced propionate. However, this needs further investigation.

The inconsistency in the *Salmonella* positive faecal samples from the infected groups with the *Salmonella* status of the ceca (at point of termination of the experiment) might highlight the importance of *Salmonella* persistence in other parts of the gut, such as colon, which requires further investigation. Irrespective of the probiotic supplementation status, the faeces of some *Salmonella* challenged chickens turned negative for *Salmonella* around week 4 post-challenge but were inconsistent in shedding profile. However, around week 8 post-challenge, more hens turned negative for *Salmonella* Typhimurium shedding in the faeces in the continuous supplemented probiotic (*n* = 5) compared with the intermittent supplemented probiotic (*n* = 2) and *Salmonella* challenged (n = 3) groups. This shows that the *Salmonella* challenged chickens could harbour the bacteria in the gut for intermittent shedding. Probiotic treatment can reduce the level of shedding but continuous or intermittent feeding of probiotics does not eliminate the pathogen.

## Conclusions

*Salmonella* Typhimurium affects the microbial abundance of certain genera that play a role in maintaining a healthy gut. Microbial genera that are increased in abundance in the *Salmonella* populated gut might play a role either in the *Salmonella* driven dysbiosis or in maintaining a normal gut function. The displaced gut microbiota can be partly restored by supplementing the feed with a *Bacillus* based probiotic, thus lowering the mean load of *Salmonella* in faeces.

## Supplementary information

**Additional file 1: Figure S1.** Rarefaction analysis of OTUs showing the quality of the reads generated from DNA obtained from chicken faeces. The flatten curves towards right show that the underlying microbial communities were well covered by the sequenced data. NC is negative control; SX is *Salmonella* challenge; CPX is continuous probiotic supplemented and *Salmonella* challenge; CPC is continuous probiotic supplemented control; IPX is intermittent probiotic supplemented and *Salmonella* challenge; IPC is intermittent probiotic control.

**Additional file 2: Figure S2.** Abundance of microbial communities at phylum level in faeces. Data for all the treatment groups were mapped in Calypso software to get the abundance of different phyla.

**Additional file 3: Figure S3.** Abundance of microbial communities at genera level in faecal samples of individual chickens in the negative control and *Salmonella* challenged chickens sampled at different time-points (days 3, 5, 7 and weeks 2, 4, 6, 8, 10 and 12 post-challenge). The genus bar is based on sampling time-points post-challenge.

**Additional file 4; Figure S4.** Faecal microbial abundance affected by *Salmonella* Typhimurium challenge at different sampling time-points in laying chickens. Panel labels (a-d) show the effect of *Salmonella* on individual bacterial genera. NC is negative control, SX is *Salmonella* challenged. Data from the faecal samples collected at days 3, 5, 7 and weeks 2, 4, 6, 8, 10 and 12 post-challenge were used for comparison between the two treatment groups (NC and SX).

**Additional file 5: Figure S5.** Microbial genera abundance of *Salmonella* turned negative chickens. The abundance level of the *Salmonella* turned negative chickens (*n* = 2) was compared with consistently *Salmonella* shedding chickens (*n* = 5) and negative control groups (*n* = 7).

**Additional file 6: Figure S6.** Microbial genera abundance affected by *Salmonella* Typhimurium challenge and continuous supplementation of probiotic. The microbial abundance at genera level of the continuous supplemented probiotic control (CPC) group was compared with the continuous supplemented probiotic and *Salmonella* Typhimurium challenged (CPX) group. Data from the faecal samples collected at nine different sampling time-points (days 3, 5, 7 and weeks 2, 4, 6, 8, 10 and 12) post-challenge were analysed for comparison between the two treatment groups (CPC and CPX).

**Additional file 7: Figure S7.** Microbial community composition and diversity affected by *Salmonella* Typhimurium and continuous supplementation of probiotic. (a) Microbial community composition between the continuous supplemented probiotic control (CPC) and the continuous supplemented probiotic and *Salmonella* Typhimurium challenged (CPX) groups. (b) Microbial diversity between the CPC and CPX at different time-points (days 3, 5, 7 and weeks 2, 4, 6, 8, 10 and 12) post-challenge. Data from the faecal samples collected at days 3, 5, 7 and weeks 2, 4, 6, 8, 10 and 12 post-challenge were used for the comparison between the two treatment (CPC and CPX) groups.

**Additional file 8: Figure S8.** Microbial abundance of individual genera affected by *Salmonella* Typhimurium challenge and intermittent supplementation of probiotic. The microbial abundance at genera level of the intermittent supplemented probiotic control (IPC) group was compared with the intermittent supplemented probiotic and *Salmonella* Typhimurium challenged (IPX) group. Data from the faecal samples collected at nine different sampling time-points (days 3, 5, 7 and weeks 2, 4, 6, 8, 10 and 12) post-challenge were analysed for comparison between the two treatment groups (IPC and IPX).

**Additional file 9: Figure S9.** Microbial community composition and diversity affected by *Salmonella* Typhimurium and intermittent supplementation of probiotic. (a) Microbial community composition between the intermittent supplemented probiotic control (IPC) and the intermittent supplemented probiotic and *Salmonella* Typhimurium challenged group (IPX). (b) Microbial diversity between the IPC and IPX at different time-points (days 3, 5, 7 and weeks 2, 4, 6, 8, 10 and 12) post-challenge. Data from the faecal samples collected at days 3, 5, 7 and weeks 2, 4, 6, 8, 10 and 12 post-challenge were analysed for comparison between the two treatment groups (IPC and IPX).

**Additional file 10: Figure S10.** Microbiota diversity and abundance of microbial genera affected by continuous supplementation of probiotic. (a) Overall diversity of faecal microbiota. (b) Abundance of faecal microbial genera. For determining the effects of the probiotic on the diversity of gut microbiota and abundance levels of individual microbial genera, the negative control (NC) group was compared with the continuous supplemented probiotic (CPC) group (excluding *Salmonella* Typhimurium challenge).

**Additional file 11: Figure S11.** Microbiota diversity and abundance of microbial genera affected by intermittent supplementation of probiotic. (a) Overall diversity of faecal microbiota. (b) Abundance of faecal microbial genera. For determining the effects of the probiotic on the diversity of gut microbiota and abundance levels of individual microbial genera, the negative control (NC) group was compared with the intermittent supplemented probiotic (IPC) group (excluding *Salmonella* Typhimurium challenge).

## Data Availability

The 16S rRNA sequence data are available from the NCBI SRA under the BioProject accession number PRJNA561675.

## Abbreviations

BPW: Buffered peptone water
BSA: Brilliance *Salmonella* agar
FDR: False discovery rate
LB: Luria-Bertani
mMPN: Miniaturised most probable number
OTUs: Operational taxonomy units
PBS: Phosphate buffered saline
RDA +: Redundancy analysis
SCFAs: Short chain fatty acids
TSS: Total sum scaling or total sum normalisation
XLD: Xylose lysine deoxycholate
