# Supplementary figures and images for: Salmonella Typhimurium infection disrupts but continuous feeding of Bacillus based probiotic restores gut microbiota in infected hens
Source: J Anim Sci Biotechnol. 2020 Mar 23;11:29. doi: 10.1186/s40104-020-0433-7 (PMC7087389; doi:10.1186/s40104-020-0433-7)

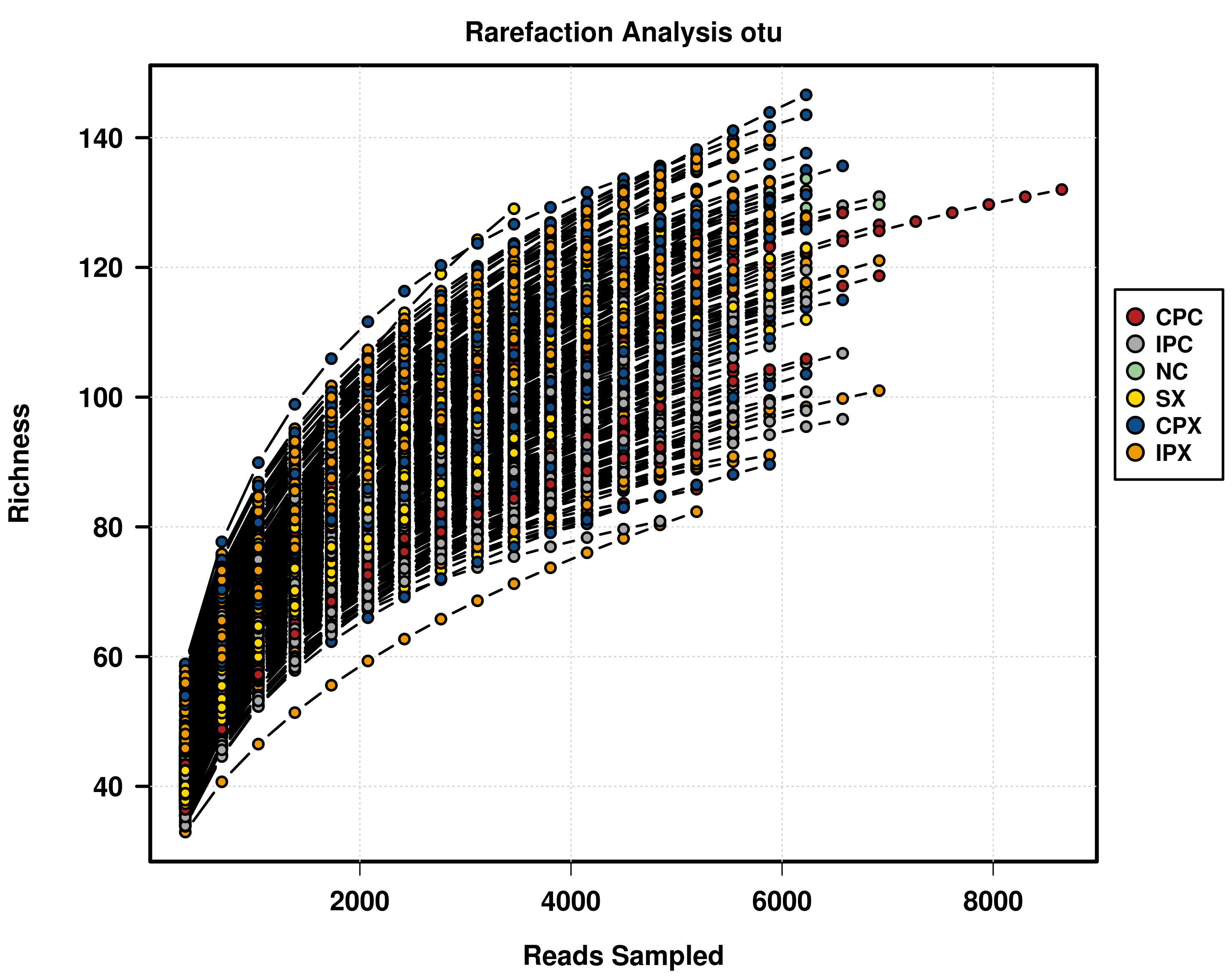

Supplement: Supplementary file 1 — Additional file 1: Figure S1. Rarefaction analysis of OTUs showing the quality of the reads generated from DNA obtained from chicken faeces. The flatten curves towards right show that the underlying microbial communities were well covered by the sequenced data. NC is negative control; SX is Salmonella challenge; CPX is continuous probiotic supplemented and Salmonella challenge; CPC is continuous probiotic supplemented control; IPX is intermittent probiotic supplemented and Salmonella challenge; IPC is intermittent probiotic control. [file 40104_2020_433_MOESM1_ESM.tif]

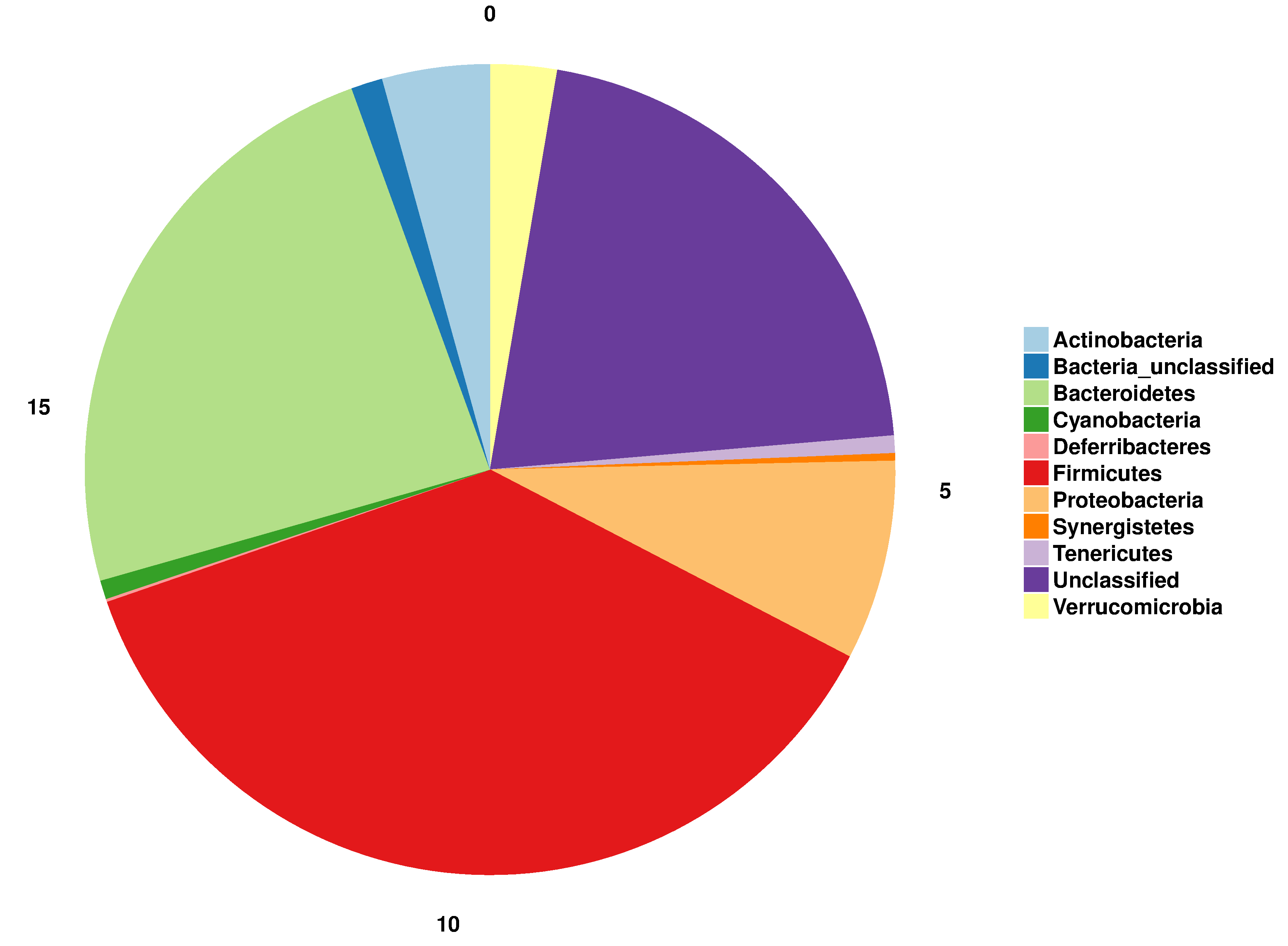

Supplement: Supplementary file 2 — Additional file 2: Figure S2. Abundance of microbial communities at phylum level in faeces. Data for all the treatment groups were mapped in Calypso software to get the abundance of different phyla. [file 40104_2020_433_MOESM2_ESM.tif]

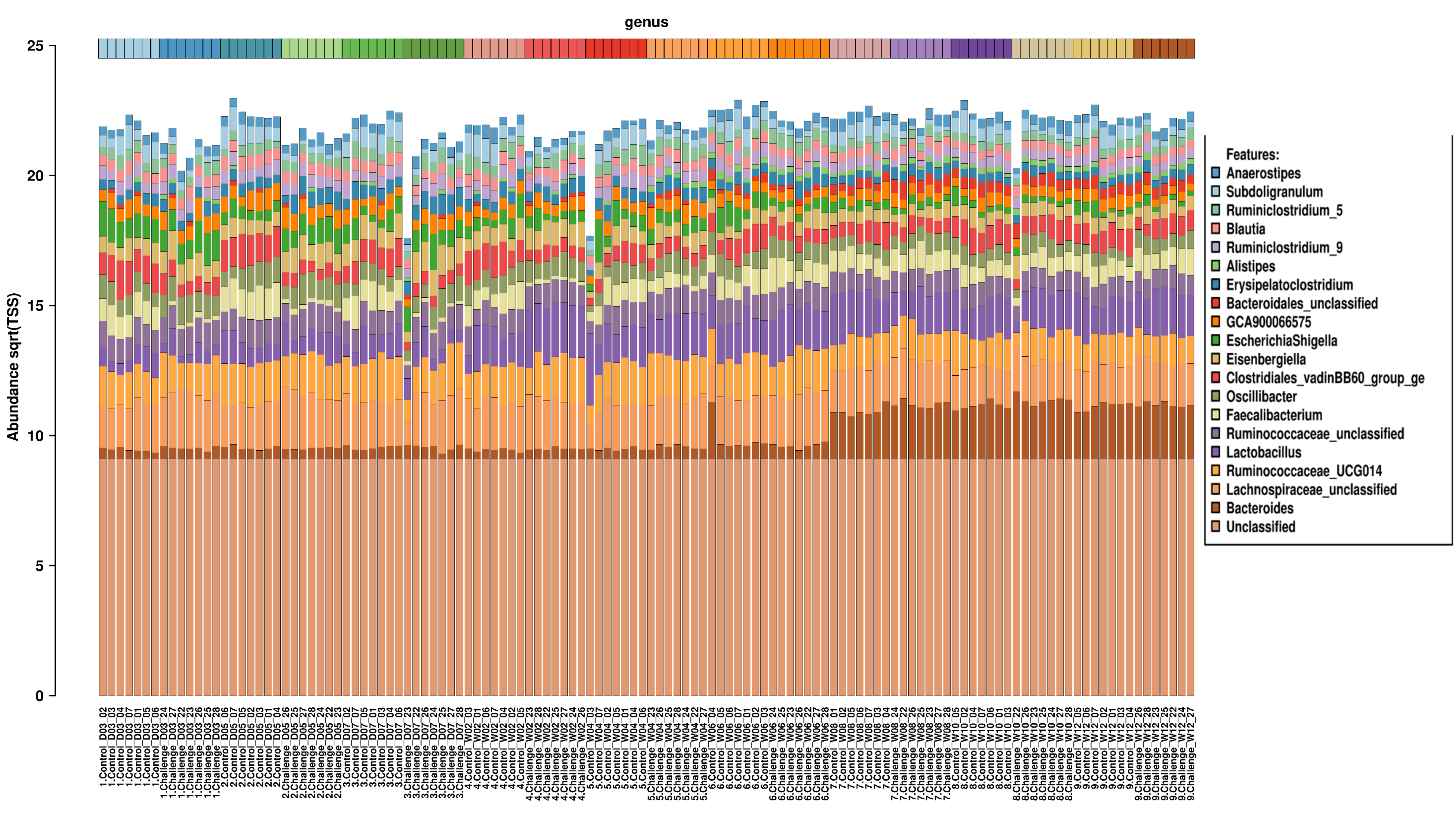

Supplement: Supplementary file 3 — Additional file 3: Figure S3. Abundance of microbial communities at genera level in faecal samples of individual chickens in the negative control and Salmonella challenged chickens sampled at different time-points (days 3, 5, 7 and weeks 2, 4, 6, 8, 10 and 12 post-challenge). The genus bar is based on sampling time-points post-challenge. [file 40104_2020_433_MOESM3_ESM.tif]

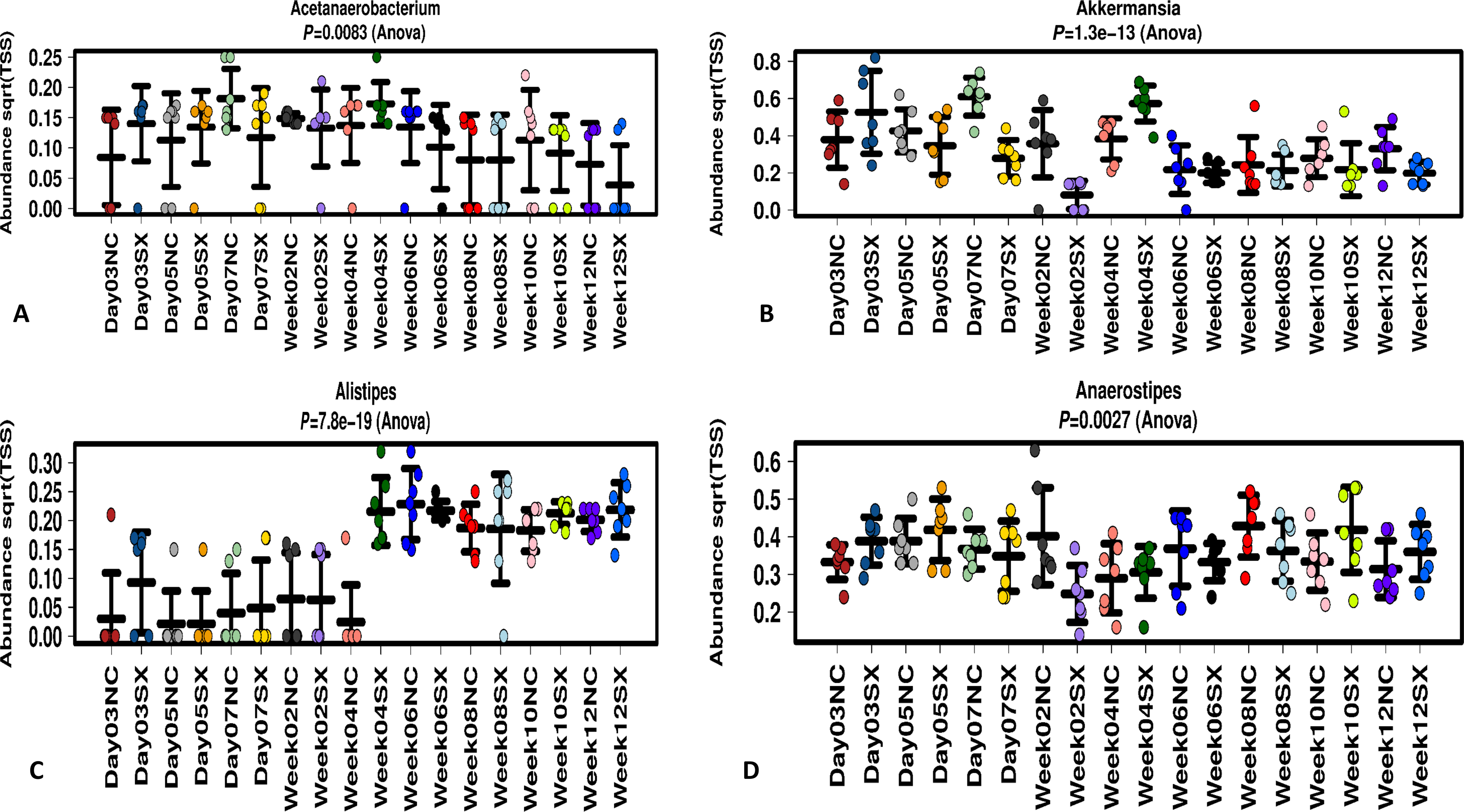

Supplement: Supplementary file 4 — Additional file 4; Figure S4. Faecal microbial abundance affected by Salmonella Typhimurium challenge at different sampling time-points in laying chickens. Panel labels (a-d) show the effect of Salmonella on individual bacterial genera. NC is negative control, SX is Salmonella challenged. Data from the faecal samples collected at days 3, 5, 7 and weeks 2, 4, 6, 8, 10 and 12 post-challenge were used for comparison between the two treatment groups (NC and SX). [file 40104_2020_433_MOESM4_ESM.tif]

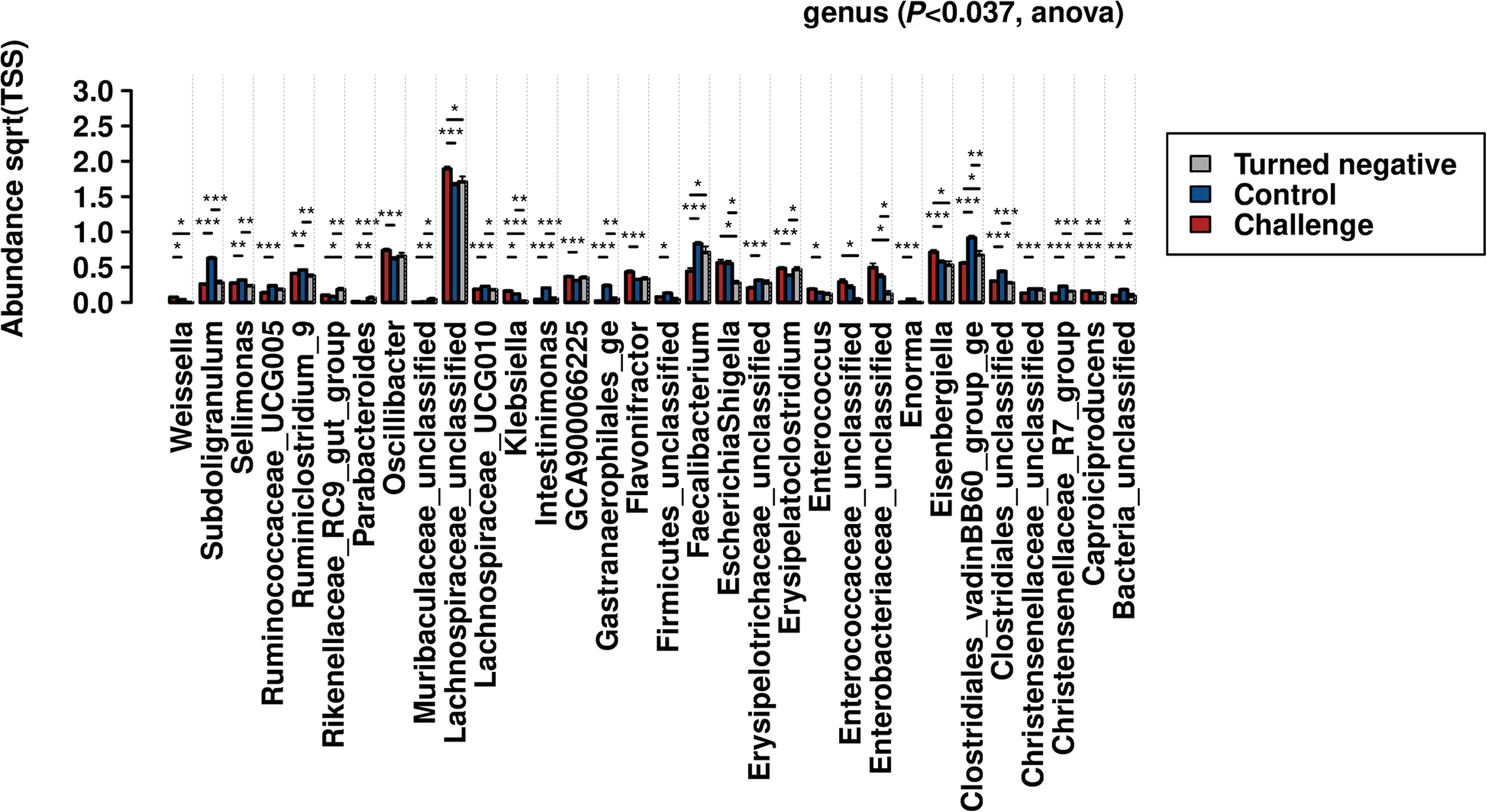

Supplement: Supplementary file 5 — Additional file 5: Figure S5. Microbial genera abundance of Salmonella turned negative chickens. The abundance level of the Salmonella turned negative chickens (n = 2) was compared with consistently Salmonella shedding chickens (n = 5) and negative control groups (n = 7). [file 40104_2020_433_MOESM5_ESM.tif]

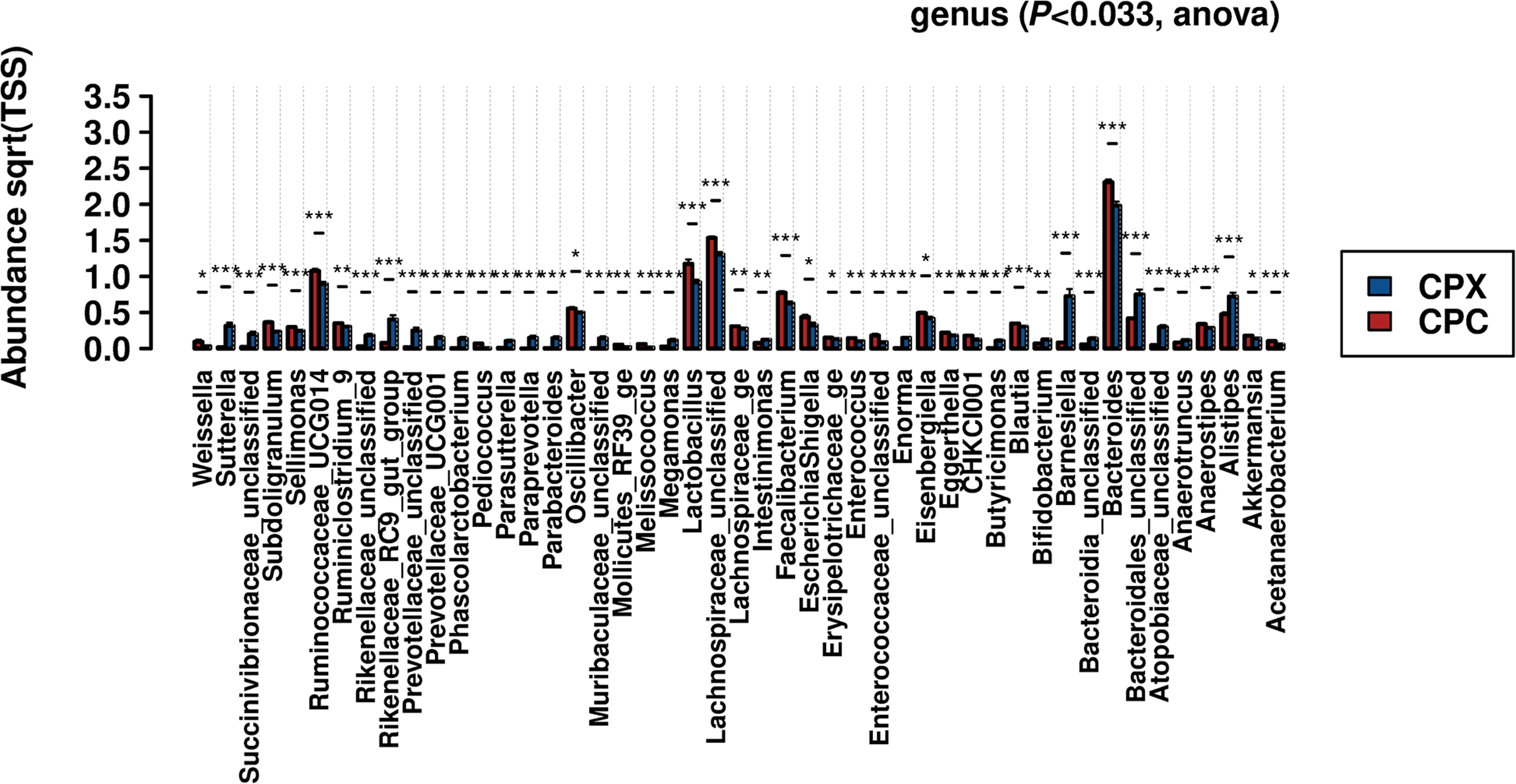

Supplement: Supplementary file 6 — Additional file 6: Figure S6. Microbial genera abundance affected by Salmonella Typhimurium challenge and continuous supplementation of probiotic. The microbial abundance at genera level of the continuous supplemented probiotic control (CPC) group was compared with the continuous supplemented probiotic and Salmonella Typhimurium challenged (CPX) group. Data from the faecal samples collected at nine different sampling time-points (days 3, 5, 7 and weeks 2, 4, 6, 8, 10 and 12) post-challenge were analysed for comparison between the two treatment groups (CPC and CPX). [file 40104_2020_433_MOESM6_ESM.tif]

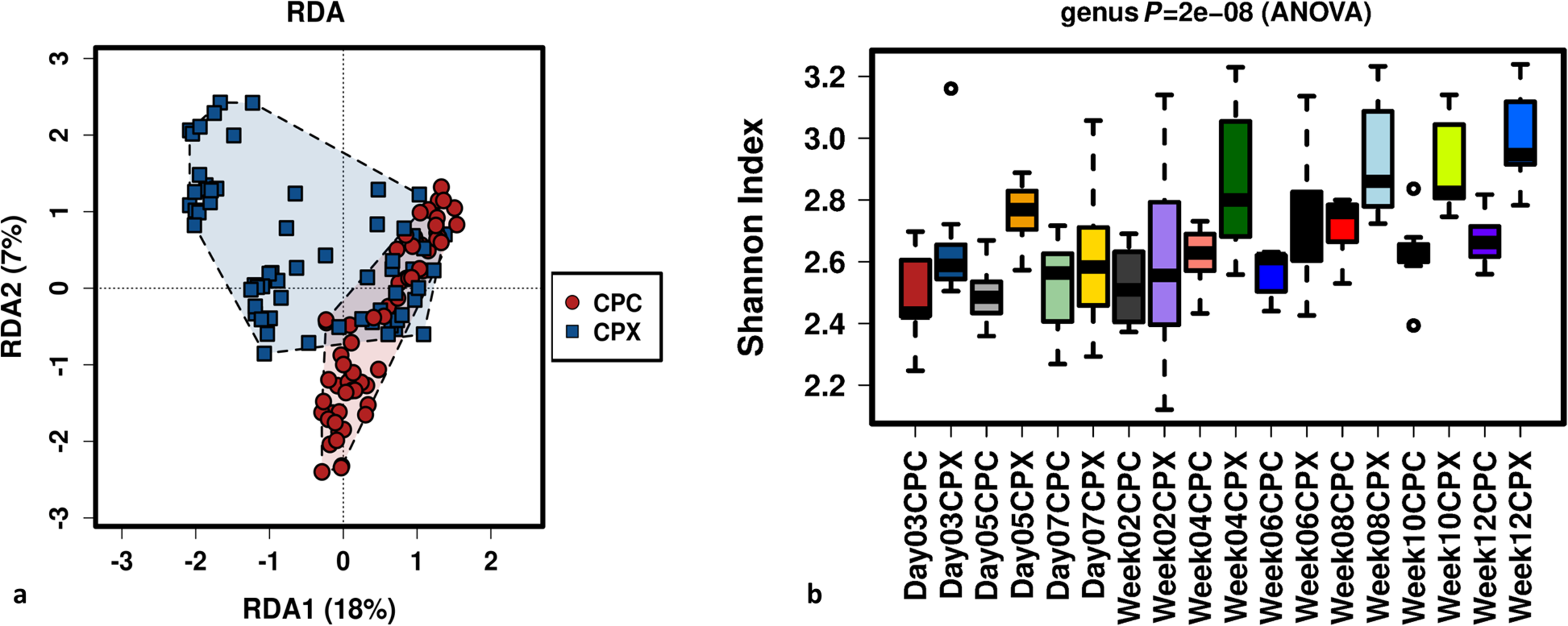

Supplement: Supplementary file 7 — Additional file 7: Figure S7. Microbial community composition and diversity affected by Salmonella Typhimurium and continuous supplementation of probiotic. (a) Microbial community composition between the continuous supplemented probiotic control (CPC) and the continuous supplemented probiotic and Salmonella Typhimurium challenged (CPX) groups. (b) Microbial diversity between the CPC and CPX at different time-points (days 3, 5, 7 and weeks 2, 4, 6, 8, 10 and 12) post-challenge. Data from the faecal samples collected at days 3, 5, 7 and weeks 2, 4, 6, 8, 10 and 12 post-challenge were used for the comparison between the two treatment (CPC and CPX) groups. [file 40104_2020_433_MOESM7_ESM.tif]

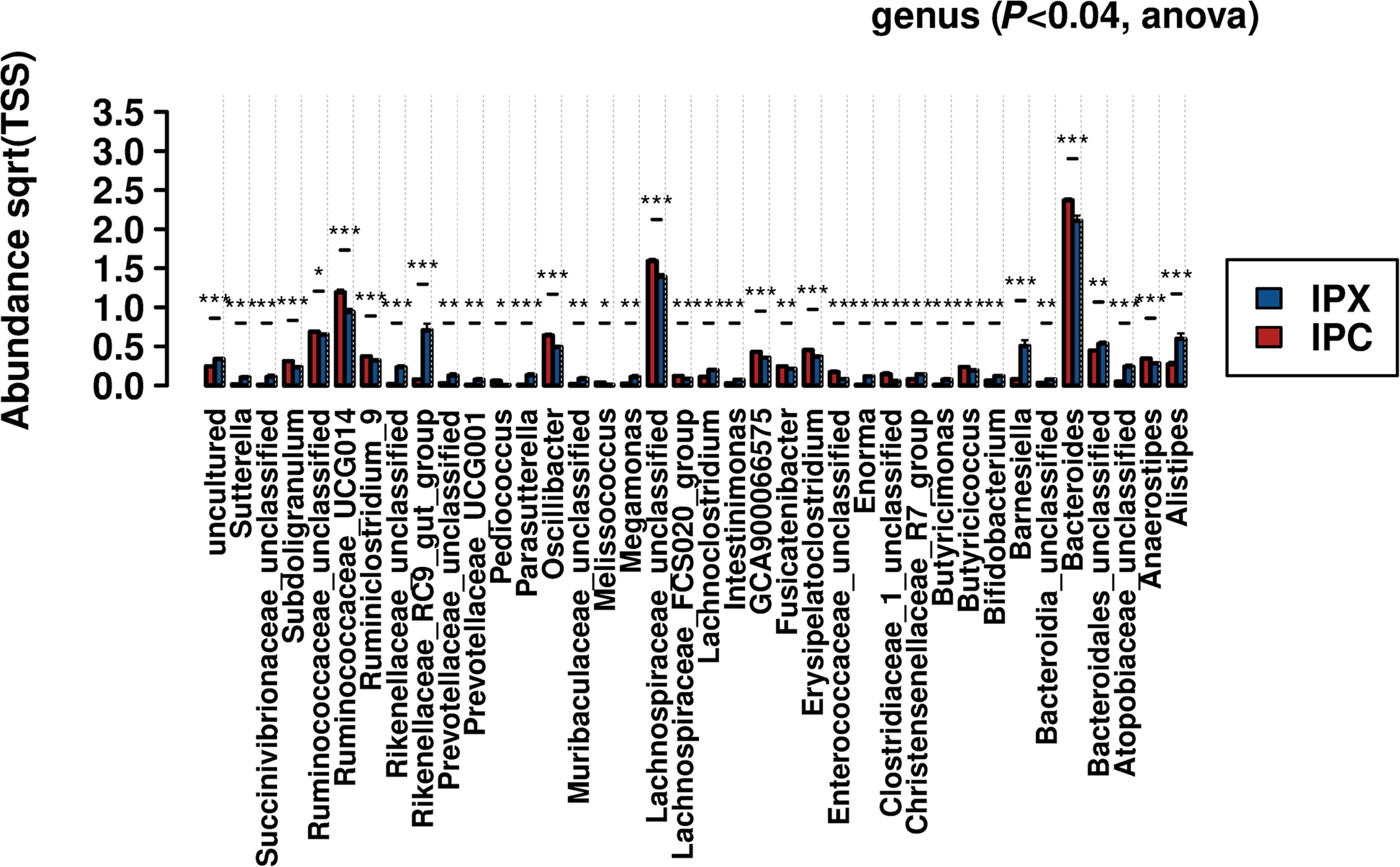

Supplement: Supplementary file 8 — Additional file 8: Figure S8. Microbial abundance of individual genera affected by Salmonella Typhimurium challenge and intermittent supplementation of probiotic. The microbial abundance at genera level of the intermittent supplemented probiotic control (IPC) group was compared with the intermittent supplemented probiotic and Salmonella Typhimurium challenged (IPX) group. Data from the faecal samples collected at nine different sampling time-points (days 3, 5, 7 and weeks 2, 4, 6, 8, 10 and 12) post-challenge were analysed for comparison between the two treatment groups (IPC and IPX). [file 40104_2020_433_MOESM8_ESM.tif]

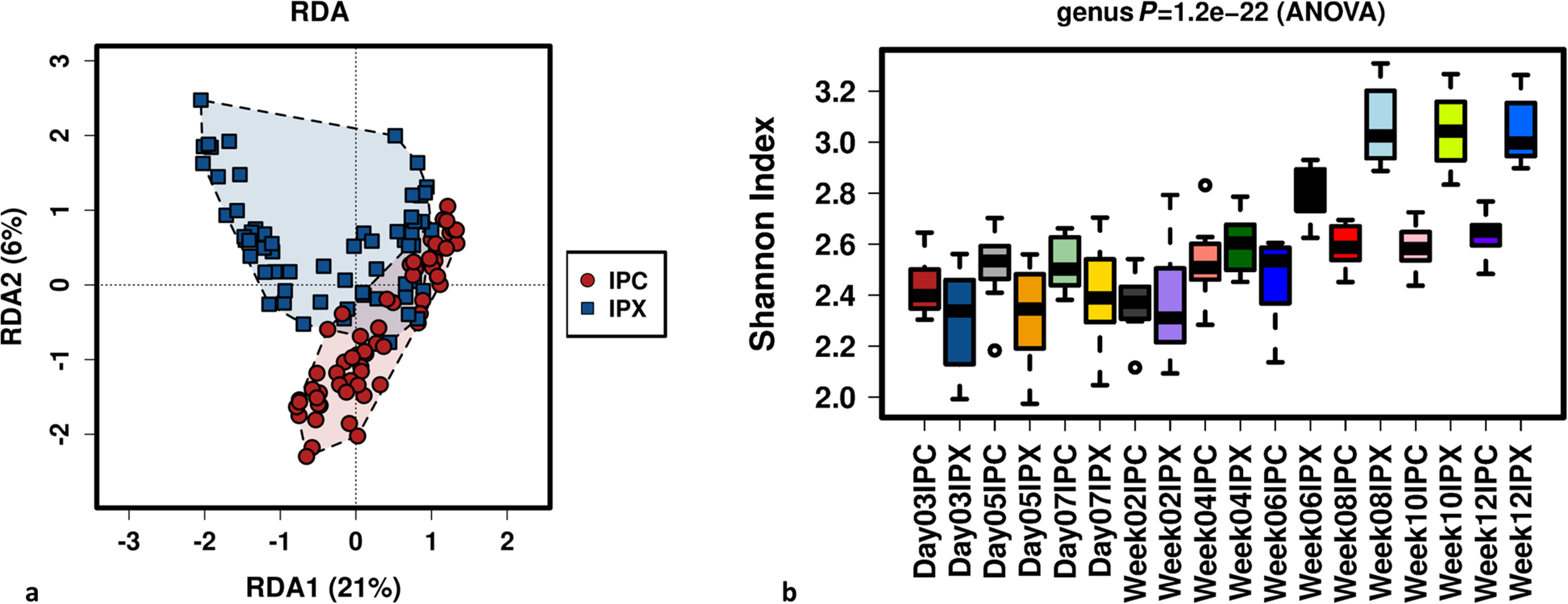

Supplement: Supplementary file 9 — Additional file 9: Figure S9. Microbial community composition and diversity affected by Salmonella Typhimurium and intermittent supplementation of probiotic. (a) Microbial community composition between the intermittent supplemented probiotic control (IPC) and the intermittent supplemented probiotic and Salmonella Typhimurium challenged group (IPX). (b) Microbial diversity between the IPC and IPX at different time-points (days 3, 5, 7 and weeks 2, 4, 6, 8, 10 and 12) post-challenge. Data from the faecal samples collected at days 3, 5, 7 and weeks 2, 4, 6, 8, 10 and 12 post-challenge were analysed for comparison between the two treatment groups (IPC and IPX). [file 40104_2020_433_MOESM9_ESM.tif]

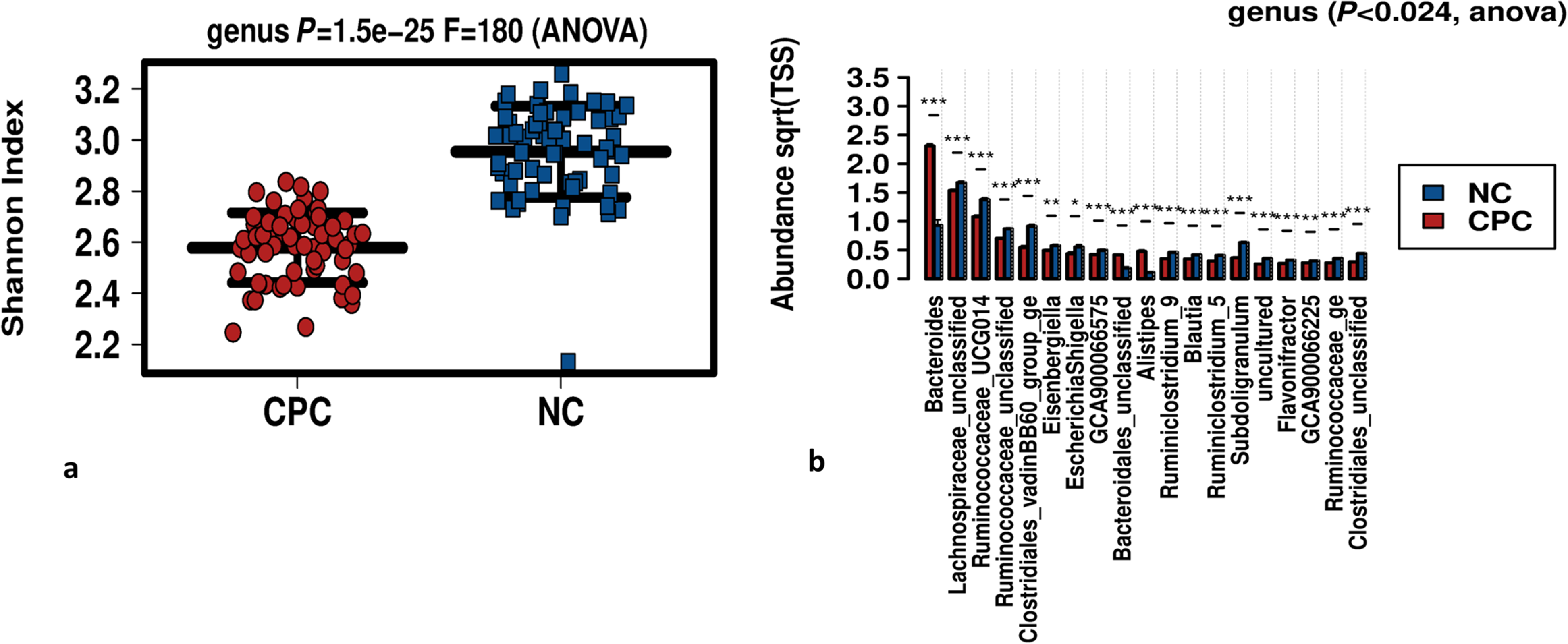

Supplement: Supplementary file 10 — Additional file 10: Figure S10. Microbiota diversity and abundance of microbial genera affected by continuous supplementation of probiotic. (a) Overall diversity of faecal microbiota. (b) Abundance of faecal microbial genera. For determining the effects of the probiotic on the diversity of gut microbiota and abundance levels of individual microbial genera, the negative control (NC) group was compared with the continuous supplemented probiotic (CPC) group (excluding Salmonella Typhimurium challenge). [file 40104_2020_433_MOESM10_ESM.tif]

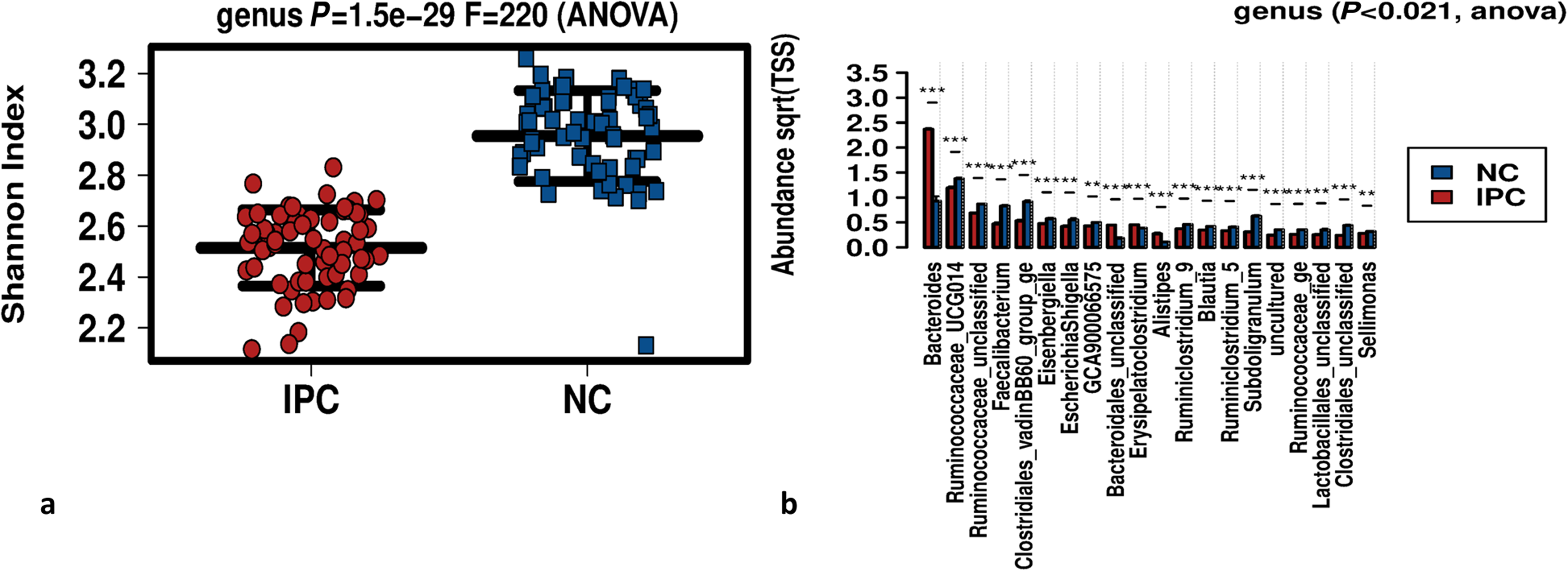

Supplement: Supplementary file 11 — Additional file 11: Figure S11. Microbiota diversity and abundance of microbial genera affected by intermittent supplementation of probiotic. (a) Overall diversity of faecal microbiota. (b) Abundance of faecal microbial genera. For determining the effects of the probiotic on the diversity of gut microbiota and abundance levels of individual microbial genera, the negative control (NC) group was compared with the intermittent supplemented probiotic (IPC) group (excluding Salmonella Typhimurium challenge). [file 40104_2020_433_MOESM11_ESM.tif]
